# Histone modification and histone modification-targeted anti-cancer drugs in breast cancer: Fundamentals and beyond

**DOI:** 10.3389/fphar.2022.946811

**Published:** 2022-09-15

**Authors:** Jianwei Feng, Xinyue Meng

**Affiliations:** Department of Ultrasound, Shengjing Hospital of China Medical University, Shenyang, China

**Keywords:** epi-drugs, histone modification, tumor suppressor gene, breast cancer, epigenetics

## Abstract

Dysregulated epigenetic enzymes and resultant abnormal epigenetic modifications (EMs) have been suggested to be closely related to tumor occurrence and progression. Histone modifications (HMs) can assist in maintaining genome stability, DNA repair, transcription, and chromatin modulation within breast cancer (BC) cells. In addition, HMs are reversible, dynamic processes involving the associations of different enzymes with molecular compounds. Abnormal HMs (e.g. histone methylation and histone acetylation) have been identified to be tightly related to BC occurrence and development, even though their underlying mechanisms remain largely unclear. EMs are reversible, and as a result, epigenetic enzymes have aroused wide attention as anti-tumor therapeutic targets. At present, treatments to restore aberrant EMs within BC cells have entered preclinical or clinical trials. In addition, no existing studies have comprehensively analyzed aberrant HMs within BC cells; in addition, HM-targeting BC treatments remain to be further investigated. Histone and non-histone protein methylation is becoming an attractive anti-tumor epigenetic therapeutic target; such methylation-related enzyme inhibitors are under development at present. Consequently, the present work focuses on summarizing relevant studies on HMs related to BC and the possible mechanisms associated with abnormal HMs. Additionally, we also aim to analyze existing therapeutic agents together with those drugs approved and tested through pre-clinical and clinical trials, to assess their roles in HMs. Moreover, epi-drugs that target HMT inhibitors and HDAC inhibitors should be tested in preclinical and clinical studies for the treatment of BC. Epi-drugs that target histone methylation (HMT inhibitors) and histone acetylation (HDAC inhibitors) have now entered clinical trials or are approved by the US Food and Drug Administration (FDA). Therefore, the review covers the difficulties in applying HM-targeting treatments in clinics and proposes feasible approaches for overcoming such difficulties and promoting their use in treating BC cases.

## Introduction

Breast cancer (BC) accounts for a highly frequent malignancy in the female population (Winters et al., 2017; Hiatt et al., 2022). According to the statistics from the World Health Organization (WHO), BC occupies 11.7% of the overall cancer patients and takes up 6% of the overall death cases. BC displays highly variable intra-tumor and inter-tumor characteristics, cancer stages when the patient is diagnosed and morphologies; as a result, it remains a challenge to effectively treat cancer and predict patient survival. In the past 10 years, BC survival shows an increasing trend due to early screening and improvement in treatment, but its 10-year survival remains unsatisfactory (80%) (Caplan, 2014). In China, a study finds differences between high-income nations and China, which discovers that the Chinese are associated with a young age at BC onset, low BC screening rate, one-child policy, delayed BC diagnosis inducing late/advanced stage when they present with symptoms, insufficient medical resources, and the low consciousness of BC (Fan et al., 2014). Consequently, it is necessary to develop new treatments. Hormone receptors (HR), in particular, progesterone receptor (PR) and estrogen receptor (ER), have important effects on BC occurrence and development (Trabert et al., 2020). Different BC subtypes are associated with different molecular and histological features, growth rates, and endocrine therapy/chemotherapy responses. Consequently, treatments are selected based on ER/PR/human epidermal growth factor receptor 2 (HER2) expression status, tumor size and grade, lymph node metastasis (LNM), and distant metastasis (DM) (Chlebowski and Anderson, 2012).

Epigenetic and genetic alterations are suggested to have a critical effect on various cell processes such as imprinting, X chromosome inactivation, chromatin remodeling, and tumorigenesis (Han et al., 2016; Yang et al., 2021a). As for epigenetic alterations, their frequently seen subtypes are histone modifications (HMs). Epigenetic alterations can be reversible, which is different from genetic mutations; as a result, they are the safer options for anti-BC treatment (Li et al., 2021). In chromatin-associated processes such as gene modulation, histone post-translational modifications (PTMs) have an essential effect, since hub histones H2A-H2B and H3-H4 are wrapped by the 147-bp DNA fragment, forming the fundamental chromatin unit (Talbert and Henikoff, 2021). HMs have been extensively studied from diverse perspectives, but it is still necessary to understand the aforementioned processes for the sake of clarifying HMs’ functions and the related enzymatic mechanisms underlying BC. Currently, over 23 classes of HMs have been identified, but just a low portion of them are associated with BC. Therefore, the present review aims to analyze histone methylation acetylation, the most extensively investigated class. Any dysregulation in the aforementioned processes induces imbalanced gene levels within BC and results in abnormalities in cell growth, migration, invasion, and treatment resistance (Byler et al., 2014; Pasculli et al., 2018).

Multidisciplinary consultation is needed in BC treatment. The most updated treatments are surgical treatment, chemotherapy, radiotherapy, and molecularly-targeted endocrine therapy, which are selected based on the BC subtype. Recently, great efforts have been made to improve targeted therapy, especially for bevacizumab-targeting vascular endothelial growth factor (VEGF) and trastuzumab (herceptin)-targeting HER2, both of which are approved (Robert et al., 2011). Epigenetic alterations have been suggested over gene mutation because of reversibility. Epigenetic modifications (EMs) are established and maintained according to special enzyme activities, histone deacetylases together with histone methyltransferases, and they are the major targets for epigenetic treatment (Qin et al., 2019a). Epigenetic treatments that use the aforementioned enzyme inhibitors can suppress tumorigenesis (Yang et al., 2021b).

The present work aims to summarize the relevant information regarding the importance of highly abundant post-translational modifications within BC, H3Kme, H4Kme, and H3Kac for BC occurrence, migration, and prognosis. Particularly, we highlight the histone marker status within BC subtypes, and the impacts on transcriptionally regulating certain genes, erasers, and writers. We also examine the effect of histone H3K and H3K-specific methyltransferase on BC and analyze the functions of histone methyltransferases (HMTs) and histone acetyltransferases (HATs) in drug-resistant cancer, together with their relevant mechanisms. Methods to diagnose and predict prognosis based on epigenetics make great contributions to precision oncology. Some approaches to diagnose DNA methylation have been applied clinically or entered clinical trials (Cowan et al., 2010). Great efforts have been made to compensate for the abnormal epigenetic mechanisms in precision oncology, which facilitate the development of epi-drugs that target epigenetic modulators. This work collects information regarding inhibitors applied in clinical trials from the ClinicalTrials.gov database maintained by the U.S. National Library of Medicine. At present, just nine epi-drugs have been approved by the FDA, including IDH, EZH2, DNA methyltransferases (DNMTs), and histone deacetylases inhibitors (HDACis). Moreover, numerous other drugs are under clinical trials for the treatment of solid tumors (NCT01928576 and NCT03179943) or hematologic tumors (NCT02717884 and NCT03164057). It is to be noted that ER-positive (ER+) BC phase-II trials (NCT00676663, NCT00828854, and NCT04190056) are conducted to test whether epi-drugs plus conventional treatments are effective, which indicates that more is known about the epigenetic mechanisms governing the development, migration, and drug resistance of ER+ BC. This section will discuss the efficacy and mechanism of action of certain DNMT and HDAC inhibitors in treating cancers.

## Histone modifications within BC

### Histone methylation in BC

**FIGURE 1:**
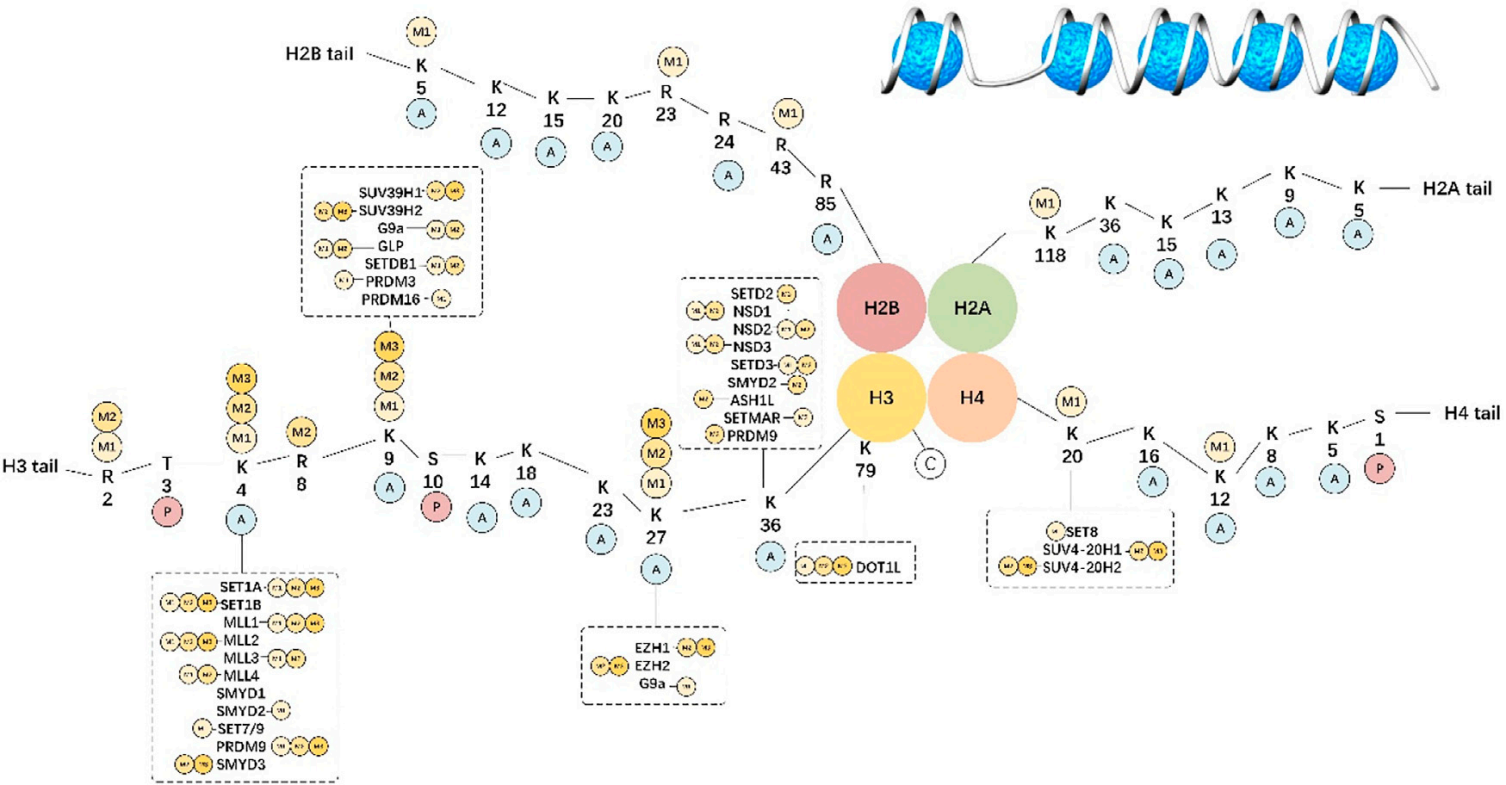
Histone methylation-specific targets.

Histone methylation may take place in arginine and lysine residues and involves complicated modifications compared with acetylation. Lysine is mono-, di-, or trimethylated, whereas arginine is asymmetrically or symmetrically methylated (Barski et al., 2007). As a reversible process, histone methylation can be strictly modulated by different demethylases (KDMs) and methyltransferases (KMTs). A portion of such markers (H3K4, H3K36, and H3K79) is related to activation at the transcriptional level, while others (H3K9, H3K27, and H4K20) are linked to suppression at the transcriptional level (Jenuwein and Allis, 2001). Figure 1 summarizes the specific targets identified for diverse HM classes.

In eukaryotic cells, chromatin is the complex formed by DNA and histones. The basic functional unit of chromatin is the nucleosome that contains a histone octamer (H2A, H2B, H3, and H4) wrapped by DNA. Histone tails undergo numerous posttranslational modifications, which are deposited by writers, removed by erasers, and read by readers, and may either loosen or tighten DNA-histone binding with active or silent transcription.

#### H3K4 methylation

H3K4 methylation shows high enrichment levels at transcriptional start sites (TSSs), promoter regions, and enhancer regions. In addition, H3K4me1 exhibits high enrichment levels in enhancer regions (Heintzman et al., 2007) and it can bind to H3K27me3 or H3K27ac, thus marking the suppressive or active enhancers, separately (Creyghton et al., 2010). Different from additional H3K4 methylation showing high enrichment levels in intergenic regions, H3K4me2 can mark the 5′-terminal in transcribed genes (Kim and Buratowski, 2009). H3K4me3 is canonically distributed in actively transcribed gene promoters and poised genes related to differentiation (Santos-Rosa et al., 2002). Set1 can form Complex Proteins Associated with Set1 (COMPASS) in yeast and is the unique enzyme related to every H3K4 methylation (Miller et al., 2001).

For mammals, the KMT2 (MLL) family is the main H3K4 HMT, which contains six members (KMT2A–D, KMT2F, and KMT2G). In addition, within human cells, six Set1 homologies (SET1A–SET1B and MLL1–MLL4) together with five additional H3K4 methyltransferases (SMYD1–SMYD3, SET7/9, and PRDM9) have also been identified (Wang et al., 2015). Moreover, the KMT2 family is classified into three categories according to the containing domain type, including KMT2A–KMT2B (MLL1–MLL2), KMT2C–KMT2D (MLL3–MLL4), together with KMT2F–KMT2G (SETD1A–SETD1B) (Shilatifard, 2008). As revealed by *in vitro* research, the core complexes of MLL1–MLL2 display mono-, di-, and low tri-methylation activities in cells (Patel et al., 2008). For instance, MLL1 is suggested to be involved in H3K4 methylation within MCF-7 cells in the estrogen-mediated transcription of ER target genes (Jeong et al., 2011). MLL1 is frequently duplicated or over-expressed within BC cells, and as a result, it may be the therapeutic target for BC treatment (Tate et al., 2019). Additionally, MLL1 can accelerate the transcription of TFF1 (the estrogen-dependent gene) by H3K4me1/2 in the enhancer region’s CpG islands and maintains the permissive chromatin architecture to bind to estrogen receptor α (ERα) and the pioneer factor (FOXA1). It results in the relaxation of chromatin for facilitating ERα binding together with its transcription within BC Jeong et al. (2014) H3K4 methyltransferase has been increasingly suggested to participate in BC occurrence. MLL2 shows a certain interaction with ERα and modulates the level of its target, thus mediating BC occurrence (Mo et al., 2006). As reported by Natarajan *et al.*, MLL2 upregulation within BC cells was related to tissue malignancy; meanwhile, MLL2 protein upregulation was also detected in tissues from patients with breast invasive carcinomas (Natarajan et al., 2010). MLL3, a protein with high mutation frequency within BC cells, is also the main factor that regulates the ERα level (Gala et al., 2018). According to recent reports, the upregulation of SETD1A and MLL3 increases the ERα level, thus supporting the growth of tamoxifen-resistant BC. Moreover, according to genome-wide research on histone methylation, MLL3 plays an essential role in H3K4 monomethylation and H3K27 acetylation within the ERα enhancer (Kim et al., 2020). MLL4 and the H3K27 demethylase UTX (KDM6A) synergistically regulate BC growth and migration (Kim et al., 2014). Jin *et al.* analyzed SETD1A’s effect on tamoxifen-resistant BC. They suggested that SETD1A increased H3K4 methylation and made the chromatin region accessible to ERα targets within ER+ BC cells to activate the ER+ targets, thereby promoting the recruitment of ERα. They further discovered that SETD1A-regulated genes overlapped with specific tamoxifen-resistant genes within ER+ BC cells, which indicated the possible relation of SETD1A with tamoxifen resistance (Jin et al., 2018). SETD1A protein expression in cells increases in other BC subtypes, such as ER+, HER2+, and triple-negative breast cancer (TNBC) relative to healthy breast cells.

SMYD2 upregulation can modulate TNBC development, which predicts dismal patient survival (Li et al., 2018). SMYD3 can upregulate WNT10B (an oncogene) expression while promoting epithelial–mesenchymal transition (EMT), thus facilitating the metastasis of BC (Hamamoto et al., 2006; Fenizia et al., 2019). SET7/9 stabilizes the ER by methylating ER K302 residue, which then effectively recruits and trans-activates target genes to enhance BC occurrence (Subramanian et al., 2008). Additionally, SET7/9 deficiency promotes the cancer stem cell (CSC) features of BC while accelerating EMT, and it is associated with disease resistance, which indicates the tumor suppressor role of SET7/9 within BC (Montenegro et al., 2016).

As reported by Montenegro et al., SETD7 suppressed EMT by upregulating cadherin-1 while downregulating epidermal growth factor receptor (EGFR) and vimentin protein expression (Montenegro et al., 2016). It was evidenced by the overexpression of SETD7 within triple-negative, metastatic MDA-MB-231 cells, downregulation through siRNAs, and inhibited activity by exposing to 50 µM (R)-PFI-2 for a 3-day period within the non-metastatic estrogen receptor α (ERα/ESR1)-positive MCF-7 cells. Furthermore, SETD7 silencing within MCF-7 cells triggered the CSC phenotype (CD44+/CD24-/low) and mammosphere de-differentiation related to cadherin-1 deficiency. Such results conformed to the greater metastatic ability of MCF-7 xenografts after SETD7 silencing (Takemoto et al., 2016).

#### H3K9 methylation

H3K9 methylation, in particular H3K9me2 and H3K9me3, is usually related to heterochromatin formation and gene suppression (Bannister et al., 2001). Apart from these, H3K9me1 can be expressed around active gene-related TSSs as well (Vavouri and Lehner, 2012). H3K9me1 and H3K9me2 show nuclear and cytoplasmic localization within mammalian cells, whereas H3K9me3 displays nuclear localization only (Towbin et al., 2012). Additionally, histones can be distributed within the cytoplasm before their chromatin assembly due to the action of histone chaperones. Actually, H3K9 shows co-translational mono- and dimethylation by SETDB1 after it is bound to ribosomes (Rivera et al., 2015). Thereafter, cytoplasmic H3 and K9me1 are assembled in the chromatin, and the product is utilized for reinforcing heterochromatin and H3K9me3 as the substrate.

**FIGURE 2:**
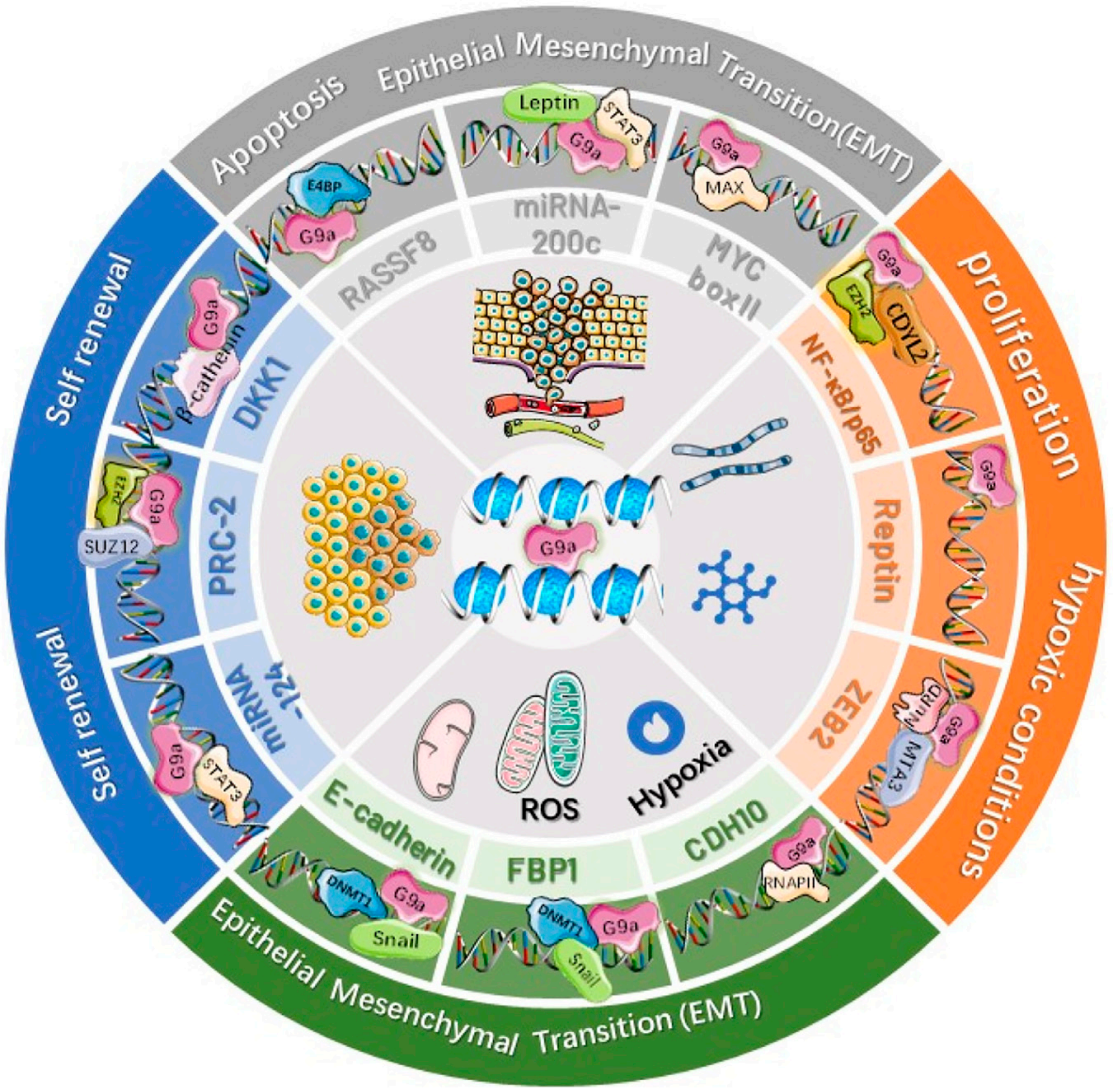
Dysregulation of G9a in various breast cancers.

Proteins belonging to the SUV39 family of human beings, including SUV39H1 (KMT1A), SUV39H2 (KMT1B), SETDB1 (KMT1E), SETDB2 (KMT1F), G9a-like protein (GLP1), and G9A (EHMT2), possess the pre-SET (N-SET) and post-SET (C-SET) domains in addition to the SET domain, which can regulate the methylation of H3K9 (Dillon et al., 2005; Wu et al., 2010). Additionally, G9a may produce homodimers or heterodimers for catalyzing H3K9me1 together with H3K9me2 within the euchromatin (Tachibana et al., 2002). In the heterochromatin, such as the pericentromeric regions, SUV39H1 can catalyze H3K9me2, while SUV39H2 catalyzes H3K9me3 (Rea et al., 2000). G9a plays a critical part in BC occurrence (Jin et al., 2022) as shown in Figure 2. Its activation can suppress anticancer genes, thereby promoting BC cell growth and migration. The overexpression of G9a can inhibit hephaestin (HEPH), thus promoting carcinogenesis of BC (Wang et al., 2017a). Additionally, G9a activation can upregulate T-Box2 (TBX2) within BC cells (Crawford et al., 2019). TBX2 overexpression promotes BC cell growth by decreasing p21WAF1 and Cdkn2a (p14Arf and p19Arf within human beings) gene expression. Suppressing G9a expression can downregulate the TBX2 level while suppressing cancer cell growth. As discovered by Zhang et al., G9a suppression induced autophagy by modulating AMPK/mTOR pathways within BC cells (Zhang et al., 2017).

Upregulation of G9a causes mono‐ or di‐methylation to lysine 9 residue of histone 3 (H3K9), resulting in an increase in the expression of TBX2, FBP1, PRC-2, and NF-κB and a decrease in the expression of DKK1, MYC, CDH10, Reptin, CASP1, ZEB2, RARRES8, and E‐cadherin in BC. G9a‐mediated up‐ and downregulation of various genes promotes cell proliferation, invasion, and metastasis and suppresses apoptosis in breast cancers.

Furthermore, SUV39H2 expression is significantly upregulated within basal-like BC, which predicts dismal BC prognostic outcomes (Liu et al., 2015a). Nonetheless, SUV39H2 mutations are detected within BC, suggesting the possibility of polymorphism within BC (Ozdag et al., 2006). TCGA-based bioinformatics analysis was carried out; as a result, SUV39H2, together with additional new genes (DNMT3B, SUV39H1, AURKB, and EZH2) was remarkably upregulated within TN disorders, which was positively related to Ki67 upregulation, tumor grade, and TN status. SUV39H2 upregulation predicted a poor survival time (Pena-Llopis et al., 2016). In ERα-positive cells, ERβ downregulates SUV39H1 and SUV39H2, and decreases the binding of ERα to p53 to abolish the suppressive heterochromatin. At last, ERβ can produce a p53-ERα transcriptional block while further suppressing proliferation and promoting apoptosis (Lu and Katzenellenbogen, 2017).

#### H3K27 methylation

H3K27 methylation has been frequently recognized as the gene repression hallmark. H3K27me3 can generate extensive domains within the silenced gene promoters (Margueron and Reinberg, 2011). Additionally, H3K27me3 is enriched at poised enhancers along with a low level of H3K4me1 in mouse and human embryonic stem cells (ESCs) (Rada-Iglesias et al., 2011). Due to the upregulation in enhancers and promoters, H3K27me3 has a critical effect on suppressing development-related genes. Apart from upregulation in poised enhancers, H3K27me2 also shows a relation to promoters in repressive and active genes (Barski et al., 2007). Unlike H3K27me2 and H3K27me3, H3K27me1 is distributed at the actively transcribed gene promoters. The PRC2 complex, which can catalyze H3K27 methylation, has four key subunits (Ezh2, Suz12, EED, and RbAP46/48) and shows the preferential methylation of H3K27. Meanwhile, G9a represents the HMT for H3K9me1/me2, which can promote H3K27 monomethylation (Coward et al., 2018).

Some previous immunohistochemical (IHC) studies have identified the relation of H3K27me3 upregulation with luminal A-like tumors. By contrast, H3K27me3 is in a low level in highly proliferative TNBC, basal-like, ER-positive, and luminal B tumors (Holm et al., 2012; Healey et al., 2014). It is interesting that H3K27me3 is related to EZH2 upregulation in TNBC and basal-like BC, indicating the role of enhanced EZH2 activity in functions associated with non-H3K27 methylation, such as specifically regulating ubiquitination and transcription factors (TFs), and protein decomposition inducing tumor genesis and development (Park et al., 2021).

#### H3K36 methylation

H3K36 methylation within human cells can interact with transcriptional elongation and methylation of H3K9 to maintain the repressive chromatin status after gene transcription in a histone acetylation-independent manner (Fang et al., 2010). Additionally, H3K36me3 can recruit DNA methyltransferase 3A (DNMT3A) for achieving DNA methylation, which is the redundant pathway for inhibiting the false initiation of transcription (Dhayalan et al., 2010). H3K36me2, which is located in gene body regions, remains largely unclear. Nonetheless, H3K36me2 upregulation is suggested to be related to aberrant transcription (Kuo et al., 2011). H3K36 methylation is able to suppress the enzymatic activity of the PRC2 complex, thus preventing PRC2-regulated H3K27 methylation (Yuan et al., 2011). Within mammalian cells, nine H3K36 methyltransferases are discovered, among which, SMYD2, NSD1-3, SETMAR, SETD3, and ASH1L directly catalyze H3K36 mono- and dimethylation, while just testis-specific PRDM9 and SETD2 are able to catalyze H3K36me3 (Eram et al., 2014).

Jeong *et al.* reported that NSD3 played an important role in epigenetically regulating BC stemness, metastasis, and EMT, indicating its role as a therapeutic target for metastatic BC (Jeong et al., 2021). Other findings indicate that SETD2 alteration-mediated epigenetic modulation and downstream H3K36me3 are involved in the development of breast phyllodes tumor (PT). In PT pathogenesis, SETD2 mutations possibly take place in the early stage (Tsang et al., 2021).

#### H3K79 methylation

H3K79 methylation shows a high enrichment level within coding regions, which is related to active transcription. H3K79 methylation occurs in the globular domains of histone H3, which is different from additional histone marks present in unstructured histone tails (Nguyen and Zhang, 2011). The aforementioned three H3K79 methylation types involve yeast Dot1 protein and the mammalian homolog DOT1L (Jones et al., 2008). It is intriguing that H3K79 methylation displays trans-tail histone modification with additional histone marks such as H4K16ac and H2B ubiquitination. In yeast, H2B ubiquitination loss reverses H3K79me2 and H3K79me3 (Ng et al., 2002). As revealed by *in vitro* HMT and structural assays, H2B ubiquitination plays an essential role in DOT1L’s methyltransferase activity (McGinty et al., 2008). H4’s N-terminal tail is needed for the *in vitro* enzymatic activity of Dot1. Furthermore, H4K16ac upregulation promotes *in vivo* H3K79 methylation (Altaf et al., 2007), and the latter has been suggested to disrupt transcriptional elongation, DNA damage response, and telomeric silencing (Huyen et al., 2004).

#### H4K20 methylation

H4K20 methylation represents the suppressive hallmark for histone modification. H4K20me1 is located in the coding region of lowly transcribed genes, which can be enriched within parental nucleosomes during cell division (Sato et al., 2016). H4K20me1/me2 can recruit leucine-rich repeats and WD repeat domain containing 1 (LRWD1) and origin recognition complex subunit 1 (ORC1) in the replication origin for regulating DNA replication (Kuo et al., 2012). It is to be noted that H4K20 methylation can directly recruit L3MBTL1 (a chromatin remodeler protein) for inducing chromatin condensation (Boccuni et al., 2003). SET8 contributes to the mono-methylation of H4K20, and later H4K20me1 is methylated into H4K20me2/me3 gradually *via* the action of SUV4-20H1/H2 (Jorgensen et al., 2013). Additionally, H4K20 methylation has been suggested to facilitate DNA damage repair, genomic stability, nucleosome turnover, DNA replication, and chromatin compaction (Tardat et al., 2010; Yang et al., 2016). The status of histone methylation marks were mesmerized in Table 1.

**TABLE 1:** Status of histone methylation marks studied in breast cancer subtypes.

Substrates	Genes	Cooperators	Cell line/Tissue	Targets	H3Kme status	Effects	References
H3K36	MLL1	—	MCF-7 breast cancer cells	↑CpG-rich region of TFF1 enhancer	H3K4me3	↑Proliferation	Jeong et al. (2014)
	MLL2	—	MDA-MB-157 and MDA-MB-231			↑Invasion	Natarajan et al. (2010)
	MLL2	ERα	MCF7 cells	↑IL-20	H3K4me1/2	↑Proliferation	Su et al. (2016)
	MLL2	GCN5	UACC812 cell line, MDA-MB-361, T47D cell lines, BT-474 cell, MCF-HER2 and MCF-Neo cell lines	↑c-Myc	H3K4me3	↑Lapatinib resistance	Matkar et al. (2015)
	MLL2	LSD1	MCF7 cells	↑NCOA3	H3K4me3	↑Proliferation	Park et al. (2016a)
				↑RSP6KB1			
	MLL3	SET1A	Tamoxifen-resistant breast cancer	↑ESR1 gene	H3K4me3	↑ERα expression	Kim et al. (2020)
	MLL3	ER	MCF7 cells	↑HOXB9	H3K4me3	↑Proliferation	Deb et al. (2016)
	MLL3	FOXA1, and ER	MCF7 cells	↑TFF1	H3K4me1	↑Proliferation	Jozwik et al. (2016)
				↑PGR			
				↑MYC			
	MLL3	—	SKBR3, BT-474, Cama-1, T47D, MCF10A HCC1954 and MDA-MB-231 MDA-MB-468 and HCC1806 cell lines	↑AGR3	H3K4me1	↑Proliferation	Gala et al. (2018)
				↑PGR			
				↑CA2			
	MLL3	promoter region of Ras genes	tamoxifen-resistant ER-positive breast cancer cells	↑PI3K/AKT/mTOR signaling pathway	H3K4me1	↑Proliferation	Wu et al. (2020)
					H3K4me3		
	SETD1A	—	MDA-MB231, MCF7, MDA-MB-468	↑SKP2	H3K4me3	↑Proliferation	Tajima et al. (2019)
						↓Senescence	
	SETD1A	—	MDA-MB-231, MCF7, BT549, and SUM159	↑MMPs	H3K4me3	↑Invasion	Salz et al. (2015)
						↑Migration	
	SET7	GATA1	MCF7, ZR75-1 and MDA-MB-231	↑VEGF	H3K4me1	↑Vascular endothelial cell proliferation	Zhang et al. (2016)
						↑Migration	
						↑Tube formation	
	SMYD3	SMAD3	MDA-MB-231 cell line	↑SNAIL1	H3K4me3	↑EMT	Fenizia et al. (2019)
	SMYD3	MRTF-A	MCF7	↑MYL9	H3K4me2/3	↑Migration	Luo et al. (2014)
H3K79	G9a	SNAIL	basal-like breast cancer	↓FBP1	H3K9me2	↑CSCs	Dong et al. (2013a)
		DNMT1					
	G9a	—	Luminal A Type Breast Cancer	↑BMP5 Expression	H3K9me2	↑Smad protein phosphorylation	Jin et al. (2022)
	G9a	EZH2	MCF7, BT474 cells, MCF7 dominant-negative TBX2 cells (MCF7-DN)	↑expression of T-Box2 (TBX2)	H3K9me3	↑NDRG1	Crawford et al. (2019)
	G9a	—	MCF7 cells	↑modulation of AMPK/mTOR pathways	H3K9me1 and H3K9me2	↓autophagy *via* AMPK	Zhang et al. (2017)
	G9a	HDAC1 and YY1	MCF-7, MDA-MB-231, MDA-MB-468, MDA-MB-435, ZR-75-30 and T47D	↑HEPH promoter	H3K9me2	↑iron homeostasis through the repression of ferroxidase hephaestin	Wang et al. (2017a)
	G9a	—		↓CDH10	H3K9me2	↑EMT	Casciello et al. (2020)
	G9a	MYC	MDA-MB-231	↓CDKN1A	H3K9me2	↑Proliferation	Tu et al. (2018)
				↓HMOX1			
				↓VAMP4			
	G9a	—	MCF7 and MDA-MB-231 (MDA231)	↓ARNTL	H3K9me2	↑Proliferation	Casciello et al. (2017)
				↓GATA2		↑Migration	
	G9a	EZH2	MCF7 and MDA-MB-231 cells	↓miR124	H3K9me2	↑Invasion	Siouda et al. (2020)
						↑EMT	
	G9a	TBX2	MCF7 and BT474 cells	↓NDRG1	H3K9me2/3	↑Proliferation	Crawford et al. (2019)
		HP1					
		EGR1					
	G9a	E4BP	MCF-7, T47D, and BT-549 cell	↓RASSF8	H3K9me2/3	↑Proliferation	Karthik et al. (2018)
		SUV39H1				↓Apoptosis	
	G9a	HDAC1	MCF-7, MDA-MB-231, S1, SK-BR-3 and MDA-MB-435	↓Hephaestin	H3K9me2	↑Proliferation	Wang et al. (2017a)
		YY1					
	G9a	STAT3	MCF12A, and MCF7	↓miR-200c	H3K9me2	↑EMT	Chang et al. (2015)
						↑CSCs	
	G9a	—	MCF-7, SKBr3, and HCT116 cells	↓LC3-II	H3K9me2	↓Autophagy	Kim et al. (2013a)
				↓GFP-LC3-II			
				↓GFP			
	G9a	SNAIL	BLBC cells and luminal cells	↓E-cadherin	H3K9me2	↑Migration	Dong et al. (2013a)
		DNMT				↑EMT	
	G9a	—	MCF-7 cells	↓Beclin-1	H3K9me2	↓Autophagy	Park et al. (2016b)
	SUV39H1	SNAIL	MCF10A, HMLE and SUM1315 cells	↓E-cadherin	H3K9me3	↑Invasion	Dong et al. (2013b)
						↑Migration	
						↑EMT	
	SUV39H2	LSD1	MDA-MB157 and MDA-MB231 cell		H3K9me3	↑Metastatic biology	Piao et al. (2015)
						↑Poor survival	
	SUV39H2	ERβ represses the expression of SUV39H1/2	MCF7 and MDA-MB-157 cells	↑transcription activated by p53	H3K9me3	↑Proliferation	Lu and Katzenellenbogen, (2017)
						↓Apoptotic activities	
	SUV39H2	Recruited by PR to methylate histone H3K9		Unknown		stabilization of HP1γ binding	Liu et al. (2014)
	SUV39H2	γ-H2AX	MCF-7, SK-BR-3, ZR-75-1, T-47D, MDA-MB-231, and BT-20	Unknown	H3K9me3	↑Chemoresistance of cancer cells	Vougiouklakis et al. (2018)
	SETDB1	SMAD3	NMuMG and MDA‐MB‐231	↓SNAIL1	H3K9me3	↓Invasion	Du et al. (2018)
						↓Camptothecin resistance	
						↓EMT	
H3K79	EZH2	Unknown	primary human breast cancer samples or xenograft tumors	↓RAD51	H3K27me3	↓HR repair	Chang et al. (2011)
						↑Breast tumor initiating cells expansion	
	EZH2	Unknown	HCC70 and MDA-MB-468 cells	↓FOXO3	H3K27me3	↑Proliferation	Gong et al. (2016)
	EZH2	Unknown		↓ERα	H3K27me3	↑Tamoxifen resistance	Nie et al. (2019)
	EZH2	Unknown	MCF10A, MDA-MB-361, MCF7, MDA-MB-436, MDA-MB-231, BT-20, HCC1937, HCC1395, MDA-MB-468, DU4475, BT-549, SUM-159, CAL-120, CAL-148, MDA-MB-453 and SUM-185	↓GATA3	H3K27me3	↑Fulvestrant resistance	Yomtoubian et al. (2020)
						↑Proliferation	
						↑Invasion	
						↑Migration	
	EZH2	Unknown	MDA-MB-231 and MCF-7	↓KLF2	H3K27me3	↑Proliferation	Taniguchi et al. (2012)
	EZH2	LncRNA UCA1		↓P21	H3K27me3	↑Tamoxifen resistance	Li et al. (2019)
	EZH2	Unknown		↓FOXC1	H3K27me3	↑Invasion	Du et al. (2012)
						↑Migration	
	EZH2	Unknown	H16N2, HME, and MCF10A	↓E-cadherin	H3K27me3	↑Invasion	Cao et al. (2008)
	EZH2	SUZ12	T47D, MCF7, and MDA-MB231	↓RKIP	H3K27me3	↑Invasion	Ren et al. (2012)
	EZH2	Unknown	MCF-7 cells	↓miR-129-5p	H3K27me3	↑EMT	Luan et al. (2016)
						↑Adriamycin resistance	
						↑Vincristine resistance	
						↑Paclitaxel resistance	
	EZH2	Unknown	MDA-MB-231(TCHu227) and MCF7(TCHu74); MDA-MB-436, MDA-MB-453, BT474 and SKBR3	↓TET1	H3K27me3	↑Proliferation	Yu et al. (2019)
						↓Senescence	
	EZH2	Unknown	MCF-7	↓RUNX3	H3K27me3	↑Proliferation	Fujii et al. (2008)
	EZH2	Unknown	MDA MB 435	↓CIITA	H3K27me3	↓Tumor immunogenicity	Truax et al. (2012)
	EZH2	Unknown	MCF-7 and ZR-75-1	↓BIK	H3K27me3	↓Apoptosis	Si et al. (2016)
						↑Paclitaxel resistance	
	EZH2	YAP	E0771 and ZR-75-30	↓GDF15	H3K27me3	↑Migration	Wang et al. (2018)
	EZH2	Unknown	MDA-MB-468 and MDA-MB-231	↓TIMP	H3K27me3	↑Invasion	Chien et al. (2018)
						↑Migration	
	EZH2	Unknown	MDA-MB-231 and MCF7	↓WWC1	H3K27me3	↑Proliferation	Liu et al. (2018)
						↑Migration	
	EZH2	Unknown	MCF-10A, and MCF-7	↓Period2	H3K27me3	↑Invasion	Yu et al. (2018)
						↑Colony formation	
						↑Mammosphere formation	
	EZH2	LINC00511	MCF7 cells and UACC-812 and MDA-MB-231 cells	↓CDKN1B	H3K27me3	↑Proliferation	Zhang et al. (2019a)
	EZH2	LncRNA DANCR	MCF10A, MCF7, T47D, MDA‐MB‐231, and MDA‐MB‐468	↓SOCS3	H3K27me3	↑Viability	Zhang et al. (2020a)
						↑Invasion	
						↑Migration	
	EZH2	LOXL1-AS1	MDA-MB-231 and MCF7	↓miR-708-5p	H3K27me3	↑Invasion	Dong et al. (2020)
						↑Migration	
	EZH2	Unknown	MCF-7/CDDP and MDA-MB-231/CDDP cells	↓miR-381	H3K27me3	↑Proliferation	Dou et al. (2019)
						↑Cisplatin resistance	
	EZH2	Unknown	MDA-MB-231 and MDA-MB-436 cells	↓FOSB	H3K27me3	↑Proliferation	Zhang et al. (2020b)
	EZH2	YY1	MDA-MB-231 and MDA-MB-453	↓OPB	H3K27me3	↑Cell Viability	Yi et al. (2021)
						↑Migration	
	EZH2	SMYD2	T-47D, Hs 578T and MCF-7 cells	↓SIAH1	H3K27me3	↑Proliferation	Zeng et al. (2019)
				↓RASSF1		↑Invasion	
				↓AXIN2		↑EMT	
	EZH2	DDX21	MDA-MB-231 and MCF-7	↓SNAIL	H3K27me3	↓EMT	Zhang et al. (2018a)
						↓Invasion	
	EZH2	LINC01133	MDA‐MB‐231, SKBR‐3, MDA‐MB‐468, ZR‐75‐1, BT474, MCF‐7 and T47D	↓SOX4	H3K27me3	↓Invasion	Song et al. (2019)
						↓Migration	
	EZH2	macroH2A1.2	MDA-MB-468, MCF-7, MCF-10–2A, and MDA-MB-231	↓LOX	H3K27me3	↓Bone metastasis	Kim et al. (2018)

HR, hormone receptors; HER2, human epidermal growth factor receptor 2; ER, estrogen receptor; ↑, up-regulated; ↓, down-regulated.

#### Histone H2A and H2B pathways in breast cancer

Histone H2A and H2B variants are recognized as the mediators of drug resistance and also of drug sensitivity in breast cancer (Nayak et al., 2015). The histone H2A.Z depletion can also be defective in the integrity and stability of the human genome. Rangasamy *et al.* presented the molecular pathways linking H2A.Z to breast cancer and mechanisms were proposed to explain how the altered H2A.Z led to tumorigenesis (Rangasamy, 2010). However, monoubiquitination of histone H2B at lysine 120 (H2Bub1) has been shown to have key roles in transcription, DNA damage response, and stem cell differentiation (Cole et al., 2015). While globally depleted in breast cancer, H2Bub1 is selectively enriched in the coding region of certain highly expressed genes, including p53 target genes in response to DNA damage, functioning to exercise transcriptional control of these loci (Atanassov et al., 2016).

### Histone demethylation in BC

Dozens of lysine demethylases (KDMs) have been reported to date that are classified into two main groups (Cloos et al., 2006; Klose et al., 2006): the amine-oxidase type lysine-specific demethylases (LSDs) and the highly conserved Jumonji C (JmjC) domain-containing histone KDMs. KMTs and KDMs have both been implicated in oncogenesis. LSD1 can exhibit either pro-tumor or anti-tumor activity in breast cancer development, highlighting a context-dependent role in regulating different biological processes possibly by using different functional domains (Hu et al., 2019a; Fang et al., 2019). JMJD3 has been associated with breast cancer progression. Xun *et al* showed that ectopic expression of JMJD3 suppresses the stem cell-like characteristics of breast cancer cells (Xun et al., 2017).

### Histone acetylation in breast cancer

KATs can be divided into two types according to their cellular localization, namely, cytoplasmic and nuclear KATs (Han et al., 2016; Trisciuoglio et al., 2018). Among them, nuclear KATs can be further divided according to enzyme transfer mechanisms and structural homology (Table 2). There are five different families discovered to have diverse functions and targets, namely, CREB-binding protein and its paralog p300 (p300/CBP), GCN5-related N-acetyltransferase (GNAT), nuclear receptor coactivator factor (NRCF) family, and MYST (Roth et al., 2001; Fiorentino et al., 2018). For p300/CBP, there are about 100 protein substrates detected, contributing to the acetylation of non-histone and histone proteins such as tumor suppressor protein p53 (Bowers et al., 2010; Simon et al., 2016). Among KATs, the MYST family contains the greatest gene number and shows the highest diversity, which is mainly related to gene silencing and DNA repair (Voss and Thomas, 2009), including MOZ (monocytic leukemia zinc finger protein), Tip60 (Tat-interactive protein), Sas2 (something about silencing), YBF2/Sas3, and MOF. They exhibit the features of one conserved 3-terminal histone acetyltransferase (HAT) domain (that contains a binding site for acetyl-CoA), one helix-turn-helix DNA-binding domain, and one C2HC zinc finger related to HAT catalytic performance (Roth et al., 2001; Brown et al., 2016). The KAT family is associated with great variability in structural characteristics, such as chromodomains, zinc fingers, and PHD fingers (Yang and Seto, 2007).

**TABLE 2:** Classification, formal names, and aliases of HATs.

Name	Gene symbol	Alias	Protein groups
Histone acetyltransferase 1	KAT1	HAT1	Writer
K(lysine) acetyltransferase 2A	KAT2A	GCN5, GCN5L2, PCAF-b, hGCN5	Writer/reader
K(lysine) acetyltransferase 2B	KAT2B	CAF, P/CAF, PCAF	Writer/reader
CREB binding protein	KAT3A	CREBBP, CBP, KAT3A, RSTS	Writer/reader
E1A binding protein p300	KAT3B	EP300, RSTS2, p300	Writer/reader
TATA-box binding protein-associated factor 1	TAF1	KAT4, BA2R, CCG1, CCGS, DYT3, DYT3/TAF1, N-TAF1, NSCL2, OF, P250, TAF(II)250, TAF2A, TAFII-250, TAFII250, XDP	Writer/reader
TATA-box binding protein-associated factor 1 like	TAF1L	TAF2A2	Writer/Reader
General transcription factor IIIC	GTF3C4	KAT12, GTF3C4, TFIII90, TFIIIC290, TFIIIC90, TFIIICDELTA	Writer
Activating transcription factor 2	ATF2	CRE-BP1, CREB-2, CREB2, HB16, TREB7	Writer
K(lysine) acetyltransferase 5	KAT5	ESA1, HTATIP, HTATIP1, PLIP, TIP, TIP60, ZC2HC5, cPLA2	Writer
K(lysine) acetyltransferase 6A	KAT6A	MOZ, MRD32, MYST-3, MYST3, RUNXBP2, ZC2HC6A, ZNF220	Writer
K(lysine) acetyltransferase 6B	KAT6B	GTPTS, MORF, MOZ2, MYST4, ZC2HC6B, qkf, querkopf	Writer
K(lysine) acetyltransferase 7	KAT7	HBO1, HBOA, MYST2, ZC2HC7	Writer
K(lysine) acetyltransferase 8	KAT8	MOF, MYST1, ZC2HC8, hMOF	Writer
Elongator acetyltransferase complex subunit 3	KAT9	ELP3	Writer
Nuclear receptor coactivator 1	KAT13A	NCOA1, F-SRC-1, RIP160, SRC1, bHLHe42, bHLHe74	Writer
Nuclear receptor coactivator 3	KAT13B	NCOA3, ACTR, AIB-1, AIB1, CAGH16, CTG26, RAC3, SRC-3, SRC3, TNRC14, TNRC16, TRAM-1, bHLHe42, pCIP	Writer
Clock circadian regulator	KAT13D	CLOCK, bHLHe8	Writer
CSRP2 binding protein	KAT14	CSRP2BP, ATAC2, CRP2BP, PRO1194, dJ717M23.1	Writer
MHC class II transactivator	CIITA	C2TA, CIITAIV, MHC2TA, NLRA	Writer
Testis-specific chromodomain protein Y 1	CDY1	CDY, CDY1A	Writer
Testis-specific chromodomain protein Y 2	CDY2	CDY2A	Writer

#### GNAT family

DNA-wrapped surrounding histones can be accessed *via* epigenetic mechanisms such as the acetylation of histone lysine. In each histone, KATs can acetylate 10–20 lysine residues. Histone acetylation will elevate negative charges onto DNA, thereby promoting proteins associated with DNA repair, transcription, and replication to access DNA (Vo and Goodman, 2001; Unnikrishnan et al., 2010). Histone lysine acetylation has been suggested to be related to fundamental transcriptional activation commonly seen in tumor cells, in particular for K9/K11/K18/K56 onto histone H3, and K5/K8/K13K16 onto histone H4 (Berger, 2007). Such acetylation procedure can be regulated *via* lysine acetyltransferases such as p300/CBP, ORC-binding HATs, monocytic leukemia zinc finger protein (MOZ), general control of amino acid synthesis 5-like 2 (GCN5), and MYST2/KAT7 (HBO1) (Kaypee et al., 2016). GCN5 silencing inhibits MDA-MB231 cell invasion, proliferation, and migration, upregulates p21, and downregulates p-AKT, p-STAT3, E2F1, and MMP9 levels within MDA-MB231 cells relative to those treated with TGF-β1. Consequently, GCN5 is the possible downstream target of the TGF-β/Smad pathway responsible for regulating EMT within BC (Zhao et al., 2018).

#### P300/CEBP family

ERα represents the TF that binds to the growth factor (GF) and hormonal signals to be activated. Actually, ERα is extensively suggested to be acetylated post-translationally *via* the activation of coactivator p300. The persistently activated ERα is related to a higher risk of BC occurrence by promoting aberrant breast tissue development. ERα acetylation can be achieved within hinge/ligand domains in K229, K299, K302, and K303 (Wang et al., 2001). Also, in another research on atypical breast hyperplasia, the ERα acetylation level increases in lysines K266 and K268 *via* p160 and p300 coactivators (Kim et al., 2006). p300/CBP contributes to ERα acetylation and promotes cell growth within BRCA1-mutated BC cells. Cross-talk with CBP and p300 coactivators within BC cells can decrease the metastatic activity by increasing E-cadherin levels (Liu et al., 2005). H3 acetylation in the promoters of Snail, ZEB1, and ZEB2 promotes the CSC-like characteristics within BC cells (Cho et al., 2015). Metadherin (MTDH) is related to BC cell metastasis and drug resistance, which can interact with CBP, and the latter is thereby translocated into the promoter of the twist family BHLH transcription factor (TWIST) and allows for proximal H3 acetylation in the promoter (Liang et al., 2015). Certain gene mutations have been indicated to upregulate p300/CBP within BC (Tillinghast et al., 2003), which is usually related to disease relapse and chemoresistance (Xiao et al., 2011).

#### MYST family

Human males absent on the first (hMOF) deficiency can be detected within certain cancer types, and its level is the marker for disease prognosis (Cao et al., 2014). Pfister *et al.* compared the non-transformed control tissues and found that the hMOF protein and mRNA levels were significantly downregulated in primary BC. In addition, the hMOF protein level was closely related to H4K16 acetylation within each tested sample. On the contrary, hMOF expression increases within certain cancer types, which is related to HBO1 acetyltransferase responsible for forming a pre-initiation complex while initiating replication (Iizuka et al., 2006). P53 shows negative regulation on HBO1 while suppressing replication in the case of cell stresses (Iizuka et al., 2008). Moreover, HBO1 expression increases within tumor cells in comparison with healthy cells (Iizuka et al., 2009); meanwhile, its phosphorylated form functions to regulate CSC genesis within BC (Duong et al., 2013). KATs are referred to as MOZ (also known as MYST3 and KAT6A), and they can form tetrameric complexes with their paralog MORF (also known as MYST4 and KAT6B). The as-formed complexes contain two small non-catalytic subunits and bromodomain- and PHD finger-containing protein 1 (BRPF1) (Kaypee et al., 2016). The aforementioned two acetyltransferases are usually mutated within BC (Lynch et al., 2013).

### Histone deacetylation in breast cancer

Numerous histone deacetyltransferases are examined in studies to achieve favorable effects (Table 3). Sirtuin (SIRT1)-mediated ERα deacetylation within BC can decrease ERα activity and suppress BC cell growth, which is the effective method for preventing BC progression. Park *et al.* investigated SIRT2 function using Sirt2^−/−^ mammary tumor cell line (MMT) derived from the spontaneous mammary tumors in Sirt2^−/−^ mice, which identified the M2 isoform of pyruvate kinase (PKM2) as a critical target of SIRT2 (Park et al., 2016c). This result was supported by Shi *et al.* who demonstrated that the high expression of SIRT2 by IHC (IHC score >3) was downregulated in tumor tissues compared with the normal adjacent tissues in 296 patients (Shi et al., 2020). In several cell lines and human breast cancer tissues, Nakagawa *et al.* analyzed the expression of class I HDACs, including HDAC1, HDAC2, HDAC 3, and HDAC 8, and investigated which subtypes of class I HDACs were overexpressed in breast cancer. They revealed the high expression levels of these class I HDACs, and IHC results for HDAC1, HDAC2, HDAC3, and HDAC8 were positive in 17 (85%), 20 (100%), 20 (100%), and 17 (85%) of 20 breast cancer cases, respectively (Nakagawa et al., 2007). HDAC6 contributes to cancer metastasis since its upregulation increases cell motility in breast cancer MCF-7 cells and its interaction with cortactin regulates motility. HDAC6 also affects transcription and translation by regulating the heat-shock protein 90 (Hsp90) and stress granules, respectively (Saji et al., 2005). HDAC11 shows different expression levels and biological functions in different systems of the human body and is among the top 1–4% of genes overexpressed in cancers, such as breast cancer (Liu et al., 2020).

**TABLE 3:** Classification, formal names and aliases of HDACs.

Name	Gene symbol	Alias	Protein groups
Histone deacetylase 1	HDAC1	GON-10, HD1, RPD3, RPD3L1	Eraser
Histone deacetylase 2	HDAC2	HD2, RPD3, YAF1	Eraser
Histone deacetylase 3	HDAC3	HD3, RPD3, RPD3-2	Eraser
Histone deacetylase 4	HDAC4	AHO3, BDMR, HA6116, HD4, HDAC-4, HDAC-A, HDACA	Eraser
Histone deacetylase 5	HDAC5	HD5, NY-CO-9	Eraser
Histone deacetylase 6	HDAC6	CPBHM, HD6, JM21, PPP1R90	Eraser
Histone deacetylase 7	HDAC7	HD7, HD7A, HDAC7A	Eraser
Histone deacetylase 8	HDAC8	CDA07, CDLS5, HD8, HDACL1, MRXS6, RPD3, WTS	Eraser
Histone deacetylase 9	HDAC9	HD9, HDAC, HDAC9B, HDAC9FL, HDRP, MITR	Eraser
Histone deacetylase 10	HDAC10	HD10	Eraser
Histone deacetylase 11	HDAC11	HD11	Eraser
Sirtuin 1	SIRT1	SIR2L1	Eraser
Sirtuin 2	SIRT2	SIR2, SIR2L, SIR2L2	Eraser
Sirtuin 3	SIRT3	SIR2L3	Eraser
Sirtuin 4	SIRT4	SIR2L4	Eraser
Sirtuin 5	SIRT5	SIR2L5	Eraser
Sirtuin 6	SIRT6	SIR2L6	Eraser
Sirtuin 7	SIRT7	SIR2L7	Eraser
ASH1-like histone lysine methyltransferase	ASH1L	ASH1, ASH1L1, KMT2H	Reader
ATPase family, AAA domain containing 2	ATAD2	ANCCA, CT137, PRO2000	Reader
ATPase family, AAA domain containing 2B	ATAD2B	—	Reader
Bromodomain adjacent to zinc finger domain 1A	BAZ1A	ACF1, WALp1, WCRF180, hACF1	Reader
Bromodomain adjacent to zinc finger domain 1B	BAZ1B	WBSCR10, WBSCR9, WSTF	Reader
Bromodomain adjacent to zinc finger domain 2A	BAZ2A	TIP5, WALp3	Reader
Bromodomain adjacent to zinc finger domain 2B	BAZ2B	WALp4	Reader
Bromodomain PHD finger transcription factor	BPTF	FAC1, FALZ, NURF301	Reader
Bromodomain containing 1	BRD1	BRL, BRPF1	Reader
Bromodomain containing 2	BRD2	D6S113E, FSH, FSRG1, NAT, RING3, RNF3	Reader
Bromodomain containing 3	BRD3	ORFX, RING3L	Reader
Bromodomain containing 4	BRD4	CAP, HUNK1, HUNKI, MCAP	Reader
Bromodomain testis-associated	BRDT	BRD6, CT9	Reader
Bromodomain containing 7	BRD7	BP75, CELTIX1, NAG4	Reader
Bromodomain containing 8	BRD8	SMAP, SMAP2, p120	Reader
Bromodomain containing 9	BRD9	LAVS3040, PRO9856	Reader
Bromodomain and PHD finger containing 1	BRPF1	BR140	Reader
Bromodomain and PHD finger containing 3	BRPF3	—	Reader
Bromodomain and WD repeat domain containing 1	BRWD1	C21orf107, N143, WDR9	Reader
Pleckstrin homology domain interacting protein	PHIP	BRWD2, DCAF14, WDR11, ndrp	Reader
Bromodomain and WD repeat domain containing 3	BRWD3	BRODL, MRX93	Reader
CECR2, histone acetyl-lysine reader	CECR2	—	Reader
KIAA2026	KIAA2026	—	Reader
Lysine methyltransferase 2A	KMT2A	ALL-1, CXXC7, HRX, HTRX1, MLL, MLL-AF9, MLL/GAS7, MLL1, MLL1A, TET1-MLL, TRX1, WDSTS	Reader
Polybromo 1	PBRM1	BAF180, PB1	Reader
SWI/SNF-related, matrix-associated, actin-dependent regulator of chromatin, subfamily a, member 2	SMARCA2	BAF190, BRM, NCBRS, SNF2, SNF2L2, SNF2LA, SWI2, Sth1p, hBRM, hSNF2a	Reader
SWI/SNF-related, matrix-associated, actin-dependent regulator of chromatin, subfamily a, member 4	SMARCA4	BAF190A, BRG1, MRD16, RTPS2, SNF2, SNF2L4, SNF2LB, SWI2, hSNF2b	Reader
SP100 nuclear antigen	SP100	lysp100b	Reader
SP110 nuclear body protein	SP110	IFI41, IFI75, IPR1, VODI	Reader
SP140 nuclear body protein	SP140	LYSP100, LYSP100-A, LYSP100-B	Reader
SP140 nuclear body protein-like	SP140L	—	Reader
Tripartite motif containing 24	TRIM24	PTC6, RNF82, TF1A, TIF1, TIF1A, TIF1ALPHA, hTIF1	Reader
Tripartite motif containing 28	TRIM28	KAP1, PPP1R157, RNF96, TF1B, TIF1B	Reader
Tripartite motif containing 33	TRIM33	ECTO, PTC7, RFG7, TF1G, TIF1G, TIF1GAMMA, TIFGAMMA	Reader
Tripartite motif containing 66	TRIM66	C11orf29, TIF1D, TIF1DELTA	Reader
Zinc finger MYND-type containing 8	ZMYND8	PRKCBP1, PRO2893, RACK7	Reader
Zinc finger MYND-type containing 11	ZMYND11	BRAM1, BS69, MRD30	Reader

## Histone methylation-targeted anti-cancer drugs

At present, just a few selective small-molecular substances with the direct inhibition effect of active sites in specific KMT2 family protein enzymes are identified (Chern et al., 2020). Epi-drugs can restore the repressive TSGs or the aberrantly activated oncogenes to suppress BC development. In addition, epi-drugs can prevent drug resistance, increase anti-tumor therapeutic effects, and enhance the radiotherapeutic effect.

### Inhibitors that target H3K4-specific HMTs for anticancer therapy

#### MLL family inhibitors

Chern *et al.* adopted the bisubstrate strategy to prepare a focused library and identified numerous strong MLL methyltransferase inhibitors. It is to be noted that compound 16 (TC-5115) shows the highest strength and displays the 16-nM IC50 value. In the complex of MLL plus another four strong inhibitors, cocrystal structures are observed, revealing the role of such inhibitors in locking the MLL SET domain within the open, inactive conformation. Further optimizing TC-5115 can assist in developing a novel anti-MLL treatment (Chern et al., 2020).

Furthermore, MLL2 expression increases in BC cells and invasive carcinomas (Natarajan et al., 2010). Similarly, MLL4 deficiency reduces H3K4me3 expression while upregulating H3K27me3 expression within SIX1, MMP9, and MMP11 genes of MDA-MD-231 cells (Rabello Ddo et al., 2013). Based on the aforementioned findings, H3K4 methyltransferase possibly connects H3K27 acetylation and H3K4 methylation within BC cells by a certain mechanism, as evidenced by research on MLL4 levels within BC cells. Afterward, the UTX-MLL4 complex significantly promoted H3K27 acetyltransferase p300 to bind to target chromatin regions, thereby additionally increasing H3K27 acetylation while enhancing the gene activation activity of the enhancer (Kim et al., 2014).

#### Menin-MLL inhibitors

Suppressing the association of menin with HMTs is a possible new treatment. At first, macrocyclic peptidomimetic inhibitors (MCP-1) are prepared for inhibiting the interaction between menin and MLL1. Meanwhile, menin shows direct binding to MI-463 and MI-503 at the low nanomolar binding affinity, which efficiently suppresses the interaction of menin with MLL (Borkin et al., 2015). Small molecules can be used to pharmacologically inhibit the interaction between menin and MLL, which can thereby prevent *in vivo* MLL leukemia progression without affecting healthy hematopoiesis. MI-463 used in combination with auranofin (an inhibitor of thioredoxin reductase) shows synergistic effects on promoting BC cell apoptosis (Kato et al., 2020). Additionally, HO-1 has a strong induction effect, which facilitates synergistically promoting the efficacy of MI-463 and auranofin. Consequently, the combined application of menin-MLL inhibitors, such as MI-463, and auranofin can efficiently treat BC by inducing ferroptosis.

#### SMYD inhibitors

RANi-induced SMYD2 silencing within TNBC cells or AZ505 (an inhibitor of SMYD2)-mediated SMYD2 inhibition remarkably decreases *in vivo* cancer development (Li et al., 2018). SMYD2 can methylate and activate the new non-histone substrates such as the NF-κB p65 subunit and STAT3 to exert its effect, thus inducing the growth and survival of TNBC cells (Li et al., 2018). As discovered in a recent research study on BC, SMYD3 shows diverse expression levels within T47D and MCF-7 BC cell lines, which enhances cisplatin resistance of MCF-7 cells (Wang et al., 2020). In addition, SMYD3 deficiency combined with cisplatin exposure suppresses the proliferation and mitochondrial membrane potential (MMP) of cells. Based on the aforementioned findings, SMYD3 has an important effect on analyzing cancer sensitivity and resistance to cisplatin. Consequently, the SMYD3 level has an important effect on BC occurrence, while inhibiting SMYD3 is the new anti-BC treatment.

#### WDR5 inhibitors

Punzi *et al.* found that WDR5 deficiency decreased cell metastatic ability by abolishing the mesenchymal phenotype of luminal B- and TN-derived cells, thereby promoting the epithelial phenotype. In addition, TGFβ1 regulates the aforementioned process, suggesting that WDR5 is important for inducing EMT by activating TGFβ1. Furthermore, the aforementioned EMT reversion also possibly results from the drug targeting effect of WDR5, which enhances the chemosensitivity of BC cells and the paclitaxel-mediated efficacy (Punzi et al., 2019).

#### SETD1 inhibitors

As reported in a study, SETD1A activates MMP levels to modulate BC metastasis (Salz et al., 2015), and another study suggests that SETD1A amplification within mixed ductal and lobular breast cancer can upregulate the H3K4me3 marker to modulate mitosis within the mitosis and DNA damage response gene promoters. In addition, SETD1A can modulate some genes regulating DNA damage response, cell cycle, and mitosis by the promoter H3K4 methylation within LC and BC cells. SETD1A loss can trigger the efficacy of aging in suppressing tumors; as a result, SETD1A possibly has a critical effect on maintaining tumor cell growth and mitosis (Tajima et al., 2019). Furthermore, SETD1A can trigger ER+ BC cell growth and invasion by modulating genes related to cell migration and survival independent of ER. Therefore, SETD1A is essential for the development of hormone therapy resistance in BC ER independently (Jin et al., 2018). SETD1B has a similar structure to SETD1A and has a critical effect on the TNBC pathogenic mechanism and survival, no matter whether H3K4 methyltransferase is activated or not, for instance, through the formation of cytoplasmic COMPASS complexes and the regulation of ADIPOR1 *via* the BOD1 interaction (Wang et al., 2017b). A study shows that SET7/9’s effect can be achieved through negatively regulating stability *via* E2F1 and DNMT methylation. According to the aforementioned findings, SET7/9 is the biomarker utilized for predicting the invasion and treatment resistance of BC cases.

### H3K9 methyltransferase targets anti-BC drugs

#### G9A inhibitor

Accordingly, the G9A inhibitor treatment efficiently abolishes NDRG1-induced TBX2 suppression and decreases cell growth after functionally inhibiting TBX2. Based on the aforementioned results, TBX2 recruits the huge repressive complex into EGR1-responsive promoters to suppress physical growth control, thus inducing out-of-control BC cell growth (Crawford et al., 2019). A study reported the benzoxazole scaffold by virtual high-throughput screening (HTS), and the design and synthesis of 24 derivatives, which are later utilized to inhibit G9a. Following the repeated screening of anti-proliferative activity and kinase, this work found that GA001, the effective G9a antagonist that had a 1.32 μM IC50 value, triggered autophagy within MCF7 cells through AMPK (Zhang et al., 2017).

### H3K27 methylation targeting potential in anti-BC treatment

Using small inhibitory molecules or chromatin-modifying enzyme inhibitors to target tumor epigenome for releasing knockout genes from repressive status is the possible potent method to research cancer and develop new drugs. For erasing the H3K27me3 mark out of gene promoters, some methods have been used, such as directly or indirectly inhibiting EZH2, incorporating H3K27me3-recognizing synthetic TFs, applying natural anti-tumor agents, or combining with known anti-tumor agents. Nonetheless, targeting H3K27 methylation during anti-BC therapy possibly triggers treatment-induced side effects. In particular, apart from the effect of suppressing H3K27 methylation, EZH2 inhibitors can suppress EZH2 activity associated with additional effects. Consequently, applying drugs targeting H3K27 methylation should be properly validated, to overcome non-methylation-associated effects.

#### EZH2 inhibitors

**FIGURE 3:**
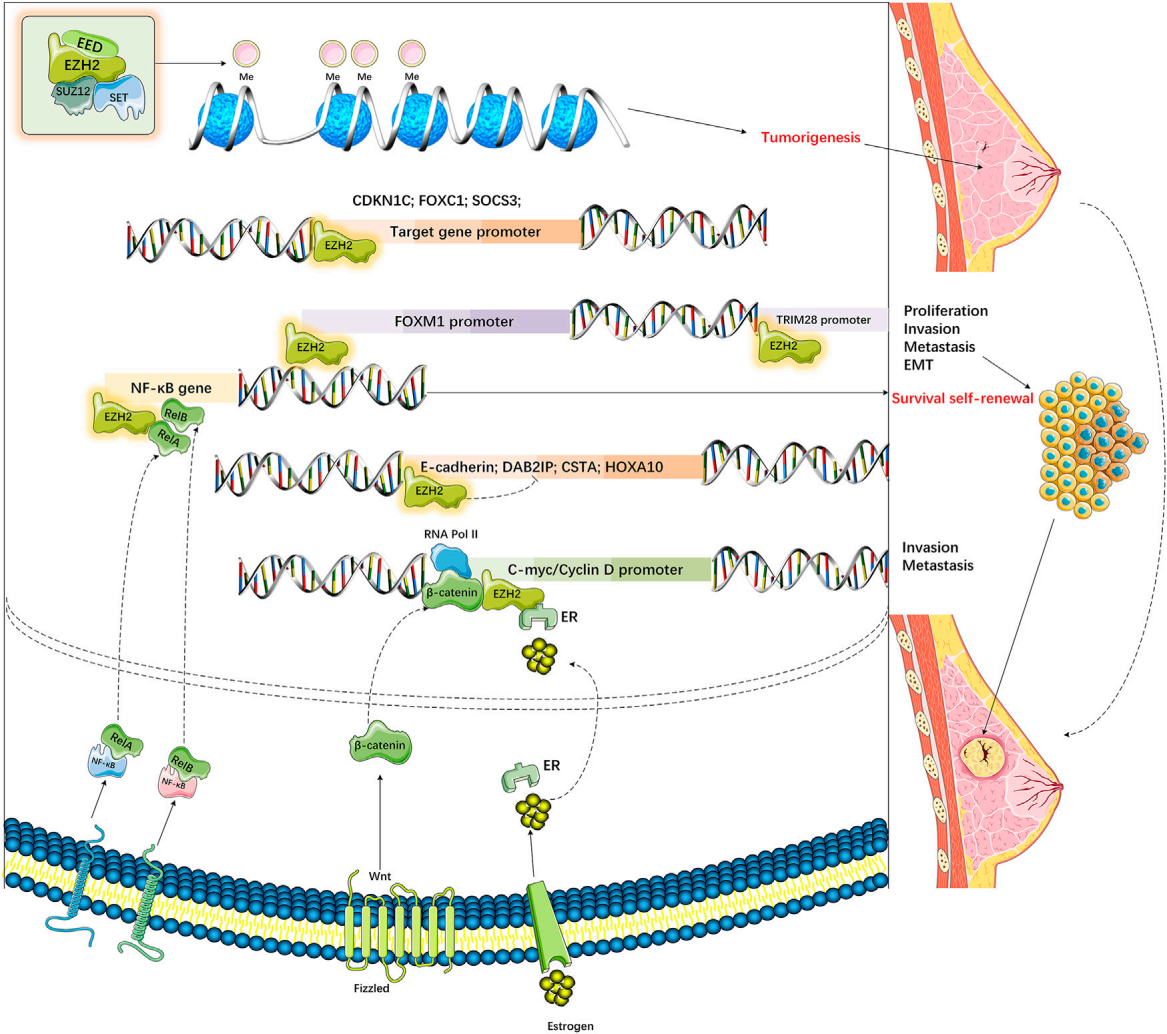
Dysregulation of EZH2 in various breast cancers.

3-deazaneplanocin A (DZNep) is a known PCR2 inhibitor that can promote tumor cell apoptosis (such as MDA-MB-468, MCF7 BC cells), but it makes no difference to healthy cells (such as MCF-10A) (Tan et al., 2007). DZNep suppresses S-adenosyl-L-homocysteine (SAH) hydrolase activity and upregulates SAH to indirectly inhibit EZH2. SAH is also an antagonist of SAM, which can thereby block HMT activity (Miranda et al., 2009). Additionally, DZNep exerts the lowest effect on DNA methylation-silenced genes (Miranda et al., 2009). DZNep treatment can markedly suppress H3K27 methylation (rather than H3K9 methylation) in diverse tumor cells (such as MB-468 BC cells) by depleting PRC2 component levels in cells (EZH2, SUZ12, and EED) (Tan et al., 2007)) (Figure 3). According to the authors, DZNep treatment reactivates PRC2-suppressed genes in BC. In BRCA1-depleted BC cells with EZH2 upregulation, DZNep further induces apoptosis compared with that in BRCA1-proficient BC cells (Puppe et al., 2009). Although DZNep has aroused wide attention as a possible antitumor therapeutic agent, there is little research on its possible side reactions *in vivo* or in a BC model. Therefore, it is necessary to carry out *in vivo* experiments to examine its use in BC as an epi-drug. After DZNep, other strong and selective EZH2 inhibitors competing against SAM have also been developed (Verma et al., 2012). Typically, GSK343 and GSK926 are suggested to down-regulate histone H3K27me3 expression while suppressing EZH2 expression within BC (HCC1806 TNBC) and PCa (LNCaP) cells; nonetheless, GSK343 displays certain limitations because it is highly cleared (plasma volume where the drug is completely eliminated per unit of time) in a rat pharmacokinetic study.

Upregulation of EZH2 causes di-methylation (H3K27me2) and H3K27 tri-methylation (H3K27me3) to lysine 27 residue of histone 3 (H3K27). EZH2‐mediated up‐ and downregulation of various genes increases cell proliferation, invasion, and metastasis and decreases the apoptosis of breast cancers.

Interestingly, PARP1, one of the poly(ADP-ribose) polymerase family (PARP) members, is suggested to decrease and interact with EZH2, thus decreasing H3K27me3 expression within MDA-MB-231 cells (Yamaguchi et al., 2018). Upon alkylation and oxidative stress-induced DNA damage, PARP1 contributes to the PARylation of EZH2 while inducing PRC2 complex dissociation, decreasing EZH2 expression, and later downregulating the expression of EZH2-regulated H3K27me3 (Quintayo et al., 2012). On the contrary, PARP inhibitor (PARPi)-mediated PARP suppression can mitigate EZH2 downregulation resulting from alkylating DNA damage, thus, further increasing EZH2-induced gene knockdown and CSC characteristics in comparison with untreated cells. Consequently, the combined application of EZH2i-like GSK343 and PARPi (olaparib) is investigated within BRCA-deficient BC (Yamaguchi et al., 2018). According to results obtained from ovarian cancer (UWB1.289) and BC (HCC38 and SUM149) cells with no response to PARPi alone, adding EZH2i enhances PARPi’s efficacy (Yamaguchi et al., 2018). Based on the aforementioned findings, it is necessary to determine whether combination therapy is effective on BRCA-defective tumors in clinical trials. Actually, a phase-II clinical trial is currently recruiting HR+/HER2− advanced BC with endocrine therapy resistance to receive SHR3162 (PARPi) and SHR2554 (EZH2i) treatment (ClinicalTrials.gov Identifier: NCT04355858).

#### H3K27 methylation inhibitors

Additionally, for BC subtypes that display the lowest H3K27me3 expression and has a dismal prognostic outcome such as TNBC, the chromatin mark may be upregulated for improving patient survival. Some studies have demonstrated the crosstalk between H3K27 methylation and additional chromatin modifications, and the combined application of HDACi (MS275) or DNMTi (guadecitabine/SGI-110) has been examined within the XtMCF and LmMCF cells, and TNBC model cells exhibiting high tumorigenicity and metastasis potentials (Su et al., 2018). The monotherapies of the aforementioned two drugs can upregulate H3K27me3 expression, whereas their combination can synergistically upregulate the H3K27me3 level. Such treatment induces transcriptional reprogramming, which is evidenced by EMT suppression, protein mutant p53 (usually detected within tumor cells), ZEB1 and EZH2 promotion, and induce E-cadherin expression, H3 trimethylation, and apoptosis. Abolishing EMT induces tumor cell proliferation, clone forming, and suppression of their stemness. Additionally, MS275 alone or plus SGI suppresses XtMCF xenograft growth, whereas MS275 decreases the lung metastasis of LmMCF cells within mice (Su et al., 2018). Collectively, the aforementioned data indicate that EMT epigenetic reprogramming, such as H3K27 methylation, inhibits TNBC cells’ aggressiveness.

## Histone acetylation targeted anti-cancer drugs

Epi-drug can suppress BC progression by abolishing the abnormally suppressed TSGs or abnormally activated oncogenes. Additionally, epi-drugs can prevent treatment resistance, increase antitumor drug efficacy, and enhance radiotherapeutic efficacy. Numerous epi-drugs are examined in clinical studies to achieve favorable effects (Table 3).

### HAT inhibitors’ effect on anti-BC treatment

Some HAT-targeting inhibitors are investigated; however, none of them has been tested in clinical trials. At present, the existing HATis are library-selected inhibitors, small-molecular HATi (either synthetic or natural), and bi-substrate inhibitors. Of them, bi-substrate mimics, including Lys-CoA, exhibit potent inhibition and are rarely applied in cells due to their great molecular weight (Lau et al., 2000; Cole, 2008). It is to be noted that most potent compound, 1r, the new compound manufactured on the basis of C646, displays potent inhibition, superior drug-like properties, and low cell proliferation after removing the toxic nitro group (Liu et al., 2019). ICG-001 can suppress BC development by targeting protein–protein interactions (PPIs) between beta-catenin and CBP, but not suppressing acetyltransferase activity (Ring et al., 2018; Sulaiman et al., 2018). HATs’ acetyltransferase activity and substrate specificity can be measured through the multi-subunit protein complexes. However, the complexities have greatly hindered the translation of *in vitro* experiments to *in vivo* ones. Existing inhibitors are poorly selective and of low efficiency, which have restricted their application, even though they are possibly efficient starting points to develop novel inhibitors.

#### KAT inhibitors (KATi)

Histone lysine acetylation is related to the occurrence and development of certain disorders, thus indicating that KAT modulators may be possible therapeutic targets. Nonetheless, it remains a challenge to identify the strong and selective KATi in comparison with modulators for additional epigenetic enzymes such as KDAC inhibitors (Merarchi et al., 2019). Some methods such as computational tools and improved assay techniques are applied in identifying small-molecular KAT inhibitors; however, just a low proportion of them are verified with *in vivo* and *in vitro* activities currently (Krishna et al., 2018; Huang et al., 2019). Such KATi are divided into three categories: *1*) bisubstrate inhibitors, *2*) natural substances and the corresponding derivatives, and *3*) synthesize small molecules.

#### Bisubstrate inhibitors

Bisubstrate analog mimicking the ternary complex constituted by the lysine substrate and cofactor acetyl-Co is the first KAT inhibitor. Thereafter, some research groups adopted the concept for identifying specific KATi. For instance, lys-CoA is prepared by connecting coenzyme A (CoA) with the single lysine residue by means of the methylene linker (Lau et al., 2000). According to reports, lys-CoA exhibits strong activity to inhibit p300 in comparison with PCAF. Additionally, H3-CoA-20 can specifically bind to PCAF (Lau et al., 2000). Additionally, Boc-C5-CoA is also suggested to occupy two binding pockets in enzyme active sites to suppress p300 (Kwie et al., 2011). Likewise, H4K16-CoA, also a bisubstrate analog, is a strong inhibitor of MYST family enzyme Tip60 and the corresponding yeast homolog Esa1, and its IC50 values are within the micromolar range (Wu et al., 2011).

Nonetheless, bisubstrate inhibitors are poorly permeable into cells and are metabolically unstable, which is ascribed to their partial peptidic structure and polar phosphate group. The aforementioned shortcomings are managed by using cell membrane penetration technologies such as lipid permeabilization and cell micro-injection (Simon et al., 2016). Some possible CoA analog prodrugs targeting p300 are also developed (Cebrat et al., 2003). Modifications including coupling the amino acid backbone of inhibitors into the arginine-abundant peptides or TAT protein transduction domain promote p300 inhibition and transmembrane delivery (Wadia and Dowdy, 2005).

According to one study, polyamine spermidine (Spd) is connected with CoA’s S atom in a covalent manner *via* the thioglycolic acid linkage, which thus forms the non-toxic Spd(N1)-CoA that can be internalized in cells by the polyamine transporter (Cullis et al., 1982). Spermidinyl-CoA-based (N1) is able to change pathways related to DNA damage repair and then enhances the chemosensitivity and radiosensitivity of cells (Bandyopadhyay et al., 2009). According to the latest article, the new peptide-CoA conjugate bisubstrate inhibitor is prepared, which displays submicromolar potential to suppress HAT1 (Ngo et al., 2019).

#### Curcumin

Many articles suggest the effect of some natural substances on inhibiting KATs (Seidel et al., 2012; Kaypee et al., 2016). Curcumin, one of the natural KATi, significantly suppresses different cancers (Shanmugam et al., 2016; Mbese et al., 2019; Tajbakhsh et al., 2018). Curcumin can suppress proliferation and clone-forming abilities of MDA-MB-231 and MCF-7 cells. Based on our results, curcumin’s inhibition against BC cells is possibly associated with its resistance to EMT and CSC properties. In line with the aforementioned results, curcumin is an anti-metastatic agent for BC (Hu et al., 2019b). New curcumin preparations have also been under investigation, such as sustained-release capsules and nanoparticles (NPs) to manage inflammation and cancer (Gupta et al., 2013; Di Costanzo et al., 2014). Curcumin can suppress pure p300’s acetyltransferase activity by adopting histone H3/p53 to be the substrate. In addition, triggering receptors expressed on myeloid cells 1 (TREM-1) within tumor-associated macrophages (TAMs) cause an inflammatory response initiated by the toll-like receptor (TLR), which shows overexpression within BC cells. TREM-1 is the risk factor for BC (Pullikuth et al., 2021). Curcumin can suppress H3/H4’s p300 acetylation to regulate TREM-1 levels within the TREM-1 promoter (Yuan et al., 2012). Moreover, curcumin is also reported to suppress KAT activity within THP-1 cells (human monocytic cell line), inhibit nuclear factor-κB (NF-κB) acetylation at Lys310, and later restrain transcription activation and nuclear translocation of the corresponding downstream targets (Yun et al., 2011). Currently, only curcumin, the KAT with the lowest specificity, is under clinical trials in the treatment of different disease (Manzo et al., 2009; Gupta et al., 2013).

#### Anacardic acid

First separated in the shell liquid of Anacardium occidentale (cashew nut), anacardic acid (also known as 6-pentadecylsalicylic acid) (Balasubramanyam et al., 2003) is identified as a non-competitive and non-selective inhibitor of PCAF and p300/CBP. It is reported to suppress Tip60 under the same experimental conditions (Ghizzoni et al., 2012). Additionally, it can also affect RelA subunit nuclear localization and acetylation to target the NF-κB pathway, thus suppressing carcinogenesis (Hemshekhar et al., 2012). Because anacardic acid is highly lipophilic and has poor physiochemical characteristics, some new phenoxyacetic acid and 6-alkyl salicylic acid analogs are analyzed to improve KAT inhibition, cell permeability, and solubility. Phenoxyacetic acid analogs have a strong KAT inhibition effect, which is decided by their alkyl chain length and location (Eliseeva et al., 2007). Additionally, anacardic acid can alleviate Tip60-induced DNA damage to augment the radiosensitivity of cancer cells (Sun et al., 2006). Moreover, changes in salicylic acid residue or alkyl chains show specific shifts in MOF suppression of MYST family KATs (Zhang et al., 2018b). Moreover, 4-cyano-3 trifluoromethylphenylbenzamides, the other anacardic acid derivative, is able to suppress KAT3 (Souto et al., 2008). Meanwhile, pentadecylidenemalonate, anacardic acid’s simplified analog, is first identified to be a KAT activator/inhibitor, which activates PCAF and suppresses recombinant CBP and p300/CBP.

#### Garcinol

Garcinol (also known as polyisoprenylated benzophenone), a strong non-specific KATi, is extracted from Garcinia indica (Kokum fruit) (Liu et al., 2015b). Its IC50 values for PCAF and p300 are 7 and 5 μM, respectively (Balasubramanyam et al., 2004). Garcinol-induced tumor cell death is related to the inhibition of cell apoptosis and histone acetylation (Arif et al., 2009). For improving garcinol’s pharmacokinetic profiles, some derivatives that have a lower toxic effect, higher efficacy, and specificity have been prepared. Isogarcinol is prepared through intramolecular cyclization, and it is adopted to be the template for designing some new KATi. In a recent study, Milite et al. prepared the benzylidene barbituric acid derivative (EML425), which was applied as a factor to selectively block p300/CBP, and had strong inhibition within the low micromolar range (IC50 values of 1.1 and 2.9 μM for CBP and p300, respectively) (Milite et al., 2015). As discovered by Ahmad et al., garcinol exposure promoted β-catenin phosphorylation, and it decreased nuclear localization in BC (Ahmad et al., 2012). Such findings were verified in the xenograft mouse model *in vivo*, in which garcinol suppressed miRNAs, NF-κB, nuclear β-catenin, and vimentin. According to the aforementioned results, garcinol’s anti-BC effect is partial because of EMT phenotypic reversal, and this is related to the abnormal levels of let-7s, miR-200s, and Wnt and NF-κB pathways to some extent.

#### Carnosol

Carnosol, which takes the region binding acetyl-CoA’s pantetheine arm, is verified to be the candidate anti-BC target. In addition, it is the new natural p300 inhibitor, which can be listed in the current inhibitor panel (Alsamri et al., 2021).

#### BET family

In recent years, BET family-targeting small molecules (BRD2–BRD4 and testis-specific BRDT) are novel epi-drugs. The BET family functions to recognize and bind to acetylated lysine by means of bromodomain; in addition, it has critical effects on cell cycle control and transcriptional elongation. At first, BET inhibitors’ effects are examined within NUT-midline carcinoma (Filippakopoulos et al., 2010) and hepatological cancers (Dawson et al., 2011; Delmore et al., 2011; Mertz et al., 2011). Thereafter, their anticancer effects are assessed in preclinical studies on additional solid tumors such as prostatic cancer (PCa) (Asangani et al., 2014), non-small cell lung cancer (NSCLC), pancreatic cancer (Garcia et al., 2016), together with BC (Ocana et al., 2017). BET inhibitors are only effective on TNBC treated combined with PLK1 inhibitors or chemotherapy or traditional treatment-resistant TNBC (Nieto-Jimenez et al., 2017). Nonetheless, as the aforementioned studies are pre-clinical studies at present, more clinical trials are needed to verify BET inhibitors’ effect on treating BC.

### Effects of HDACs inhibitors on BC therapy

HDACs can be further classified into four main types according to their sequence homology, namely, class I (HDAC1–3, and HDAC8), class II (HDAC4–7 and HDAC9–10), class III (sirtuin 1–7), and class VI (HDAC11) (Dawson and Kouzarides, 2012). The zinc metal ion is necessary for HDACs of class I/II/IV, so HDAC inhibitors can block the catalytic performance of HDACs by chelating zinc ions. Of diverse HDAC inhibitors, romidepsin and vorinostat have been approved by the FDA for the clinical treatment of cutaneous T-cell lymphoma (Olsen et al., 2007; Piekarz et al., 2009). As HDAC inhibitors display anticancer efficacy in different cancers *in vitro* and *in vivo* (Beckers et al., 2007; Kim et al., 2013b), they can be applied in clinical practice for more tumors such as BC.

According to their different structures, HDACis are classified as four types, namely, cyclic peptides, hydroxamic acids, benzamides, and aliphatic fatty acids (Table 4). At present, three HDACis of the hydroxamic acid type are approved by the FDA, which are vorinostat (SAHA), panobinostat (LBH-589), and belinostat (PXD101).

**TABLE 4:** Summary of HDAC inhibitors on the BC therapeutic strategy and corresponding clinical trials.

Drug	Therapeutic strategy	Conditions	Phases	NCT
Vorinostat (SAHA)	Monotherapy	BC	I, II (active, not recruiting)	NCT00416130
	Monotherapy	BC	II (completed)	NCT00262834
	Monotherapy	BC	I (Completed)	NCT00788112
	Vorinostat, cyclophosphamide, paclitaxel, trastuzumab, doxorubicin	Locally advanced BC	I, II (completed)	NCT00574587
	Vorinostat, carboplatin, nab-paclitaxel	Operable BC	II (active, not recruiting)	NCT00616967
	Vorinostat, paclitaxel, bevacizumab	Metastatic BC	I, II (completed)	NCT00368875
	Vorinostat, anastrozole, letrozole, exemestane	Stage Ⅳ BC	Completed	NCT01720602
	Vorinostat, trastuzumab	Metastatic or locally recurrent BC	I, II (completed)	NCT00258349
	Vorinostat, anastrozole, letrozole, exemestane	Stage Ⅳ BC	Completed	NCT01153672
	Vorinostat, radiation	BC patients with brain metastasis	I (completed)	NCT00838929
	Vorinostat, olaparib	Relapsed/refractory and/or metastatic BC	I (recruiting)	NCT03742245
	Vorinostat, tamoxifen, pembrolizumab	BC	II (terminated)	NCT02395627
	Vorinostat, tamoxifen, pembrolizumab	ER-positive BC	II (active, not recruiting)	NCT04190056
	Vorinostat, tamoxifen	Hormone therapy-resistant BC	II (completed)	NCT00365599
	Vorinostat, doxorubicin	BC	I (completed)	NCT00331955
	Vorinostat, ixabepilone	Metastatic BC	I (completed)	NCT01084057
Belinostat (PXD101)	Belinostat, ribociclib	Metastatic BC	I (recruiting)	NCT04315233
	Belinostat, talazoparib	Metastatic BC	I (recruiting)	NCT04703920
	Belinostat, trastuzumab	BC	I (suspended)	NCT03432741
Entinostat (SNDX-275)	Monotherapy	ER-positive BC	II (completed)	NCT00828854
	Monotherapy	TNBC	I (terminated)	NCT03361800
	Entinostat, exemestane	Advanced BC	II (completed)	NCT00676663
	Entinostat, exemestane	ER-positive BC	I (active, not recruiting)	NCT02820961
	Entinostat, atezolizuma	TNBC	I (active, not recruiting)	NCT02708680
	Entinostat, exemestane, atezolizumab	Hormone receptor-positive and HER2-negative BC	I, II	NCT03280563
	Entinostat, exemestane	Advanced or recurrent BC	I (active, not recruiting)	NCT02623751
	Entinostat, exemestane, goserelin	Recurrent hormone receptor-positive BC	E2112 phase III	NCT02115282
	Entinostat, nivolumab, Lpilimumab	Metastatic or locally advanced BC	I (active, not recruiting)	NCT02453620
	Entinostat, Exemestane, erlotinib	BC	I (completed)	NCT01594398
	Entinostat, capecitabine	Metastatic BC, high risk BC after neo-adjuvant therapy	I (recruiting)	NCT03473639
	Entinostat, exemestane	Hormone receptor-positive, locally advanced or metastatic BC	III (active, not recruiting)	NCT03538171
	Entinostat, exemestane	Advanced or recurrent BC	II (active, not recruiting)	NCT03291886
	Entinostat, azactidine	Advanced BC	II (active, not recruiting)	NCT01349959
	Entinostat, lapatinib, trastuzumab	Locally recurrent or distant relapsed metastatic BC	I (completed)	NCT01434303
Panobinostat (LBH-589)	Monotherapy	HER2-negative locally recurrent or metastatic BC	II (completed)	NCT00777049
	Panobinostat, letrozole	Metastatic BC	I, II (completed)	NCT01105312
	Panobinostat, paclitaxel, trastuzumab	HER2-positive or metastatic BC	I (completed)	NCT00788931
	Panobinostat, capecitabine, lapatinib	BC	I (completed)	NCT00632489
Romidepsin	Monotherapy	BC	I (active, not recruiting)	NCT01638533
	Monotherapy	Metastatic BC	II(Completed)	NCT00098397
	Romidepsin, cisplatin, nivolumab	Metastatic TNBC, BRCA mutation locally recurrent or metastatic BC	I, II (suspended)	NCT02393794
	Romidepsin, abraxane	Metastatic inflammatory BC	I, II (terminated)	NCT01938833
Valproic acid (VPA)	Valproate, hydralazine, doxorubicin, cyclophosphamide	BC	II (terminated)	NCT00395655
	Valproic acid, temsirolimus, cetuximab, bevacizuma	Recurrent BC	I (recruiting)	NCT01552434
	Valproic acid, epirubicin, 5-fluorouracil, cyclophosphamide	BC	I (completed)	NCT00246103
Ricolinostat	ACY-1215, nab-paclitaxel	Metastatic BC	I (completed)	NCT02632071
Mocetinostat	MGCD0103, docetaxel	BC	I (terminated)	NCT00511576
CUDC-101	Monotherapy	BC	I (completed)	NCT01171924

Such agents present antitumor activity in BC as well. For instance, SAHA can suppress cell growth, EMT, migration, and invasion and induce cell apoptosis, differentiation, autophagy, anoikis, and cell cycle arrest (Lee et al., 2016; Wawruszak et al., 2019; Wawruszak et al., 2021). SAHA remarkably promotes response and suppresses the resistance to tamoxifen (Lee et al., 2012), cisplatin (Wawruszak et al., 2015), olaparib (Min et al., 2015), taxol (Shi et al., 2010), epirubicin (Marchion et al., 2004), docetaxel, and trastuzumab (Bali et al., 2005). Also, SAHA efficiently promotes TNF-related apoptosis-inducing ligand (TRAIL)-mediated apoptosis, and this is achieved by triggering anoikis, increasing CD137 receptor expression, and suppressing Apo2L/TRAIL resistance (Bellarosa et al., 2012; Zhou et al., 2016). Nonetheless, SAHA can enhance TNBC cell metastasis and EMT by suppressing HDAC8, indicating that it should be cautious when treating BC with SAHA, since it might accelerate tumor metastasis (Wu et al., 2016). Meanwhile, a study showed that the combination SAHA and epigallocatechin-3-gallate (EGCG) is effective in inducing apoptosis of breast cancer cells and reducing their migratory capacity (Steed et al., 2020). In addition, Carlisi et al. showed that SAHA synergistically sensitized MDA-MB231 cells to the cytotoxic effect of parthenolide (Carlisi et al., 2015). Belinostat (PXD101) can suppress cell growth and promote cell apoptosis by PKC and Wnt/b-catenin pathways; moreover, applying belinostat in combination with the HSP90 inhibitor (17-AAG) can synergistically exert the anti-tumor effect (Lu et al., 2019; Zuo et al., 2020). Panobinostat (LBH-589) abolishes EMT in TNBC by suppressing ZEB1/2 (Rhodes et al., 2014). There are also additional HDACis of the hydroxamic acid type, including resminostat, abexinostat, and pracinostat, even though they are less tested in BC. In this regard, more research is warranted to examine whether they can be applied in BC. As discovered by Qin et al., panobinostat inhibited TNBC and non-TNBC cell growth, invasion, and migration, while promoting their apoptosis. Likewise, panobinostat suppresses BC proliferation and invasion within mouse models (Qin et al., 2019b). Romidepsin (FK2280), one of the cyclic peptide HDACi, has been approved by the FDA, and it can synergistically inhibit cell proliferation while promoting cell apoptosis when used in combination with decitabine (an inhibitor of methyltransferase) (Cooper et al., 2012). In inflammatory BC, romidepsin exposure can destroy the lymphatic vascular structure and tumor emboli by repressing HIF-1a and VEGF within inflammatory BC. Moreover, romidepsin synergistically suppresses primary tumor proliferation and multiple metastases when used in combination with paclitaxel (Robertson et al., 2013).

Valproic acid (VPA), the HDACi of the aliphatic fatty acid type, has been extensively studied, and it suppresses BC occurrence by upregulating apoptosis pathways and inducing cell cycle arrest (Fortunati et al., 2008; Travaglini et al., 2009). In addition, VPA promotes EMT by increasing ZEB1 and SNAIL levels HDAC2-dependently, but the HDAC2-related mechanism is still unknown (Zhang et al., 2019b). Additionally, VPA synergistically suppresses BC development when applied in combination with anti-tumor agents such as tamoxifen, epirubicin, cisplatin, camptothecin, and capecitabine (Marchion et al., 2005; Fortunati et al., 2008; Arakawa et al., 2009; Terranova-Barberio et al., 2016).

Entinostat (Ent, MS-275), the synthetic benzamide derivative of HDACi, exhibits potent immunomodulation on BC (McCaw et al., 2019; Connolly et al., 2021). In addition, Ent exposure can suppress EMT and the tumor-promoting cell phenotype, thereby inhibiting tumor occurrence and metastasis (Shah et al., 2014). Ent can induce the expression of retinoid acid to improve differentiation mediated by retinoic acid when it is applied together with doxorubicin and all-trans retinoic acid (ATRA); moreover, such a combination further increases doxorubicin-induced cytotoxicity. Moreover, Ent together with ATRA can manage the resistance to the aromatase inhibitor (AI) by decreasing the quantity of tumor-initiating cells (Merino et al., 2016). Additional new multifunctional inhibitors achieve favorable antitumor efficacy.

Sirtuin inhibitors suppress BC development through diverse structures, targets, and activities. In addition, they can deal with the problem of multidrug resistance through combined use with chemotherapeutics. For instance, amurensin G suppresses SIRT1 and later inhibits MDR1 and FoxO1 levels within the doxorubicin-resistant BC cells, thus potentiating doxorubicin absorption into cells and suppressing oncogenic development (Oh et al., 2010). Splitomicin can decrease cell motility while potentiating paclitaxel’s effect on resisting cell motility. Such an effect is intensified by the addition of trichostatin A (TSA), the HDAC6 inhibitor (Bonezzi et al., 2012). Some studies are conducted to evaluate SIRT1/2 inhibitors, including salermide, sirtinol, cambinol, splitomicin, nicotinamide, tenovin, suramin, indole derivatives, and analogs with similar structures. The aforementioned molecules can upregulate p53 acetylation or induce pro-apoptotic, SIRT1-epigenetically silenced gene expression to suppress BC cell growth and trigger p53-dependent apoptosis (Peck et al., 2010). Consequently, different SIRT inhibitors show synergistic effects with conventional antitumor agents in the treatment of BC. There are different pathways related to the drug resistance escape mechanism of SIRTs, indicating that more SIRT inhibitors may be prepared according to the known inhibitors for balancing efficacy and specificity. Phase-I and phase-II clinical studies have been conducted to evaluate the effects of HDAC and DNMT inhibitors on treating BC (Falahi et al., 2014). Epi-drugs exhibit poor anticancer effects on BC, and epi-drug monotherapy can just achieve an effective rate of 10% in BC cases, indicating that monotherapy may not be appropriate for treating BC. Nonetheless, according to the aforementioned clinical trials, when epi-drugs are used in combination with targeted or cytotoxic therapies, such as ER-targeted therapy, the OS and PFS are improved (Falahi et al., 2014). Consequently, the existing clinical trials mostly apply epi-drugs in combination with traditional treatments.

## Epi-drugs in clinical practice

These encouraging preclinical findings have laid the sound basis to translate epi-drugs to clinical trials for treating BC. Table 4 summarizes epi-drug-related clinical trials for the treatment of BC (mostly from https://www.clinicaltrials.gov/). Many accomplished (NCT00262834, NCT00777049, and NCT01171924) along with ongoing (NCT00416130, NCT01638533, and NCT04676516) clinical trials have been conducted to predict the safety, pharmacodynamics, and pharmacokinetics of epi-drugs for determining the best doses and monotherapy schemes. It is to be noted that the tolerance of 300/400 mg oral SAHA (twice/day for a 14-day period, separated by a 7-day rest) has been verified (Vansteenkiste et al., 2008). However, the present work just enrolled two BC cases, making it impossible to accurately determine the response rate. More clinical trials are being conducted to predict the best SAHA dose. Apart from monotherapy, epi-drugs have been frequently utilized in combination with other drugs in clinics. For instance, one phase-II trial applied SAHA plus tamoxifen in treating BC resistant to hormone therapy, and the objective response rate (ORR) and clinical benefit rate were determined to be 19% and 40%, respectively (Munster et al., 2011). Moreover, the combined application of Ent and exemestane increased the OS from 19.8 months (as obtained after exemestane monotherapy) to 28.1 months (Yardley et al., 2013). One recent phase-II clinical trial applied SAHA in combination with tamoxifen and pembrolizumab in improving the response to immunotherapy among ER+ BC cases. The treatment strategy achieved an ORR and clinical benefit rate of 4% and 19%, respectively (Terranova-Barberio et al., 2020). Typically, as revealed by an ongoing phase-II trial (NCT04190056), the aforementioned combination strategy can trigger an immune response for treating ER+ BC, while reducing the dose and adverse reactions. BETis and SAHA synergistically treat BC with olaparib in preclinical trials (Min et al., 2015; Yang et al., 2017). Additionally, there are two ongoing trials (NCT03901469 and NCT03742245) analyzing whether epi-drugs plus PARPis are effective and safe by suppressing DNA damage repair. Taken together, these clinical trials further verify the effectiveness of epi-drugs in treating BC, which should be further investigated.

## Limitation and prospects of epi-drugs in breast cancer

In this study, we reviewed histone modifications and their functions and potential cellular interactions, which might result in the development of potential efficient therapies with KATi and HDACi. Several synthetic compounds currently in pre-clinical studies have exhibited potent KATi activities against breast malignant cells. Moreover, they can effectively augment the anti-cancer activities of standard chemotherapeutic agents such as paclitaxel, doxorubicin, and cisplatin and sensitize drug-resistant cells to radiation therapy. Nevertheless, as shown, KATi and HDACi seem to be a promising group of anti-cancer drugs, particularly in combination with other anti-cancer drugs and/or radiotherapy. More large-scale promising evidence needs to be obtained from multi-center clinical trials. Meanwhile, their use in combination with other drugs and the schedule of such drug combinations need to be further investigated in both preclinical and clinical studies.

Currently, selectivity is one of the biggest challenges in developing drugs targeting epigenetic modifiers. Most currently developed drugs do not show selectivity to certain enzymes; instead, they target molecules that have certain common functions and structures. But epigenetic agents are most advantageous in their good tolerance and low severe adverse reaction rate, even though there are certain concerns about the safety of certain medicines (Cheng et al., 2019). Additionally, more reports indicate that the response rates are poor after short-term treatment, and resistance is developed in the end, which can be attributed to the transcriptional plasticity driven by epigenetics responding to environmental stress (Dawson, 2017). More multicenter and randomized phase-III studies should be conducted to realize the full potential and specificity of HDACis therapy in various subtypes of breast cancer. Further clinical studies should include the most promising novel HDACi and isozyme-specific inhibitors.

## Conclusion

The present work focuses on summarizing relevant studies on HMs related to BC and the possible mechanisms associated with abnormal HMs. Additionally, we also aim to analyze existing therapeutic agents together with those drugs approved and tested through pre-clinical and clinical trials, to assess their roles in HMs. Moreover, epi-drugs that targeted HMT inhibitors and HDAC inhibitors should be tested in preclinical and clinical studies for the treatment of BC. Epi-drugs that target histone methylation (HMT inhibitors) and histone deacetylation (HDAC inhibitors) are now under clinical trials or approved by the US Food and Drug Administration (FDA). Therefore, the review covers the difficulties in applying HM-targeting treatments in clinical applications and proposes feasible approaches for overcoming such difficulties and promoting their use in treating BC cases. Indeed, the full clinical therapeutic scope and commercial value of such agents in the field of oncology are only just emerging.

## Abbreviations

BC, breast cancer; DNMTs, DNA methyltransferases; EMs, epigenetic modifications; ERα, estrogen receptor α; ESCs, embryonic stem cells; FOXA1, pioneer factor; GF, growth factor; HATs, histone acetyltransferases; HEPH, hephaestin; HER2, human epidermal growth factor receptor 2; HMs, histone modifications; HMTs, histone methyltransferases; HR, hormone receptors; KATi, KAT inhibitors; KDMs, lysine demethylases; KMTs, lysine methyltransferases; MCP-1, macrocyclic peptidomimetic inhibitors; MTDH, metadherin; NF-κB, nuclear factor-κB; NSCLC, non-small cell lung cancer; ORR, objective response rate; PCa, prostatic cancer; PPIs, protein–protein interactions; PTMs, post-translational modifications; SIRT1, sirtuin; TCGA, The Cancer Genome Atlas; Tip60, Tat-interactive protein; TSSs, transcriptional start sites; VPA, valproic acid.
